# Physiological characteristics of IRR 400 series rubber clones ( *Hevea brasiliensis* Muell. Arg.) on drought stress

**DOI:** 10.12688/f1000research.129421.1

**Published:** 2023-01-27

**Authors:** Syarifah Aini Pasaribu, Mohammad Basyuni, Edison Purba, Yaya Hasanah

**Affiliations:** 1 Unit Research Sungei Putih, Indonesian Rubber Research Institute, Galang, Deliserdang, North Sumatra, 20585, Indonesia; 2 Department of Forestry, Universitas Sumatera Utara, Medan, North Sumatra, 20155, Indonesia; 3 Center of Excellence for Mangrove, Universitas Sumatera Utara, Medan, 20155, Indonesia; 4 Department of Agrotechnology, Universitas Sumatera Utara, Medan, North Sumatra, 20155, Indonesia

**Keywords:** rubber, drought stress, water content, adaptation, abiotic stress

## Abstract

**Background**: Drought stress is one of the main causes of plant death. Strategies for plants survival are morphological adaptations, specific signaling pathways, and tolerance mechanisms. Rubber plantations have many uses, such as foreign exchange sources, job sources, forest revitalization, and a source of alternative wood for building materials and furniture. The rubber plant’s response to drought stress is a complex biological process. A tolerant rubber clone in a dry area is the right approach. The present study aimed to determine the mechanism of drought-tolerant clones, based on physiological characteristics, to obtain character selection and drought-tolerant clones early.

**Methods**: The first factor examined for this work was clones (IRR 425, IRR 428, IRR 429, IRR 434, IRR 440, RRIC 100, and BPM 24) and the second factor was water content (30%, 60%, and 90%). The study was arranged on a factorial randomized block design and repeated three times. Characteristics observed were total sugar (µM), proline (mg/L), chlorophyll a, b, total (µg/mL), hydrogen peroxidase (µmol/g), ascorbate peroxidase (unit/mg), superoxide dismutase (unit/mg), and peroxide dismutase (unit/mg).

**Results**: The tolerance ability of the IRR 400 series rubber clones to drought stress was determined by observing the characteristics of sugar total and proline. The concentration of total sugar and proline were higher when the plant was treated with a lower water content. The selected clones tolerant to drought stress are RR 425 and IR 434 with high total sugar content and proline. Other characteristics, namely chlorophyll a, b, and total, as well as hydrogen peroxidase, ascorbate peroxidase, super oxide dismutase, peroxide dismutase, cannot be used as selection characteristics for this study.

**Conclusions:** This drought study of IRR 400 clones with varying water content percentages illustrated that the total sugar and proline characteristics could be used to distinguish tolerance levels from other observed characteristics.

## Introduction

In rubber plants, drought can cause a delayed maturation phase, short tapping period, slow latex flow, dry latex, increased dry tapping grooves, and even tree death. ^1^ Drought is one of the main abiotic stresses that affects plants and can reduce yield and productivity in almost all plants in the world. ^2^ Hence, it becomes most important compared with other environmental factors because it interferes with plant growth and development and disrupts production and performance. Water is part of the protoplasm and makes up 85–90% of the total weight of the plant tissue. Water is a vital reagent in photosynthesis and hydrolysis reactions. In addition, it acts as a solvent for salts, gases and other substances transported between cell tissues to maintain cell growth and leaf shape stability. ^3^

One of the primary sources of natural rubber is found in the Amazon basin, South America. ^4^ Optimal conditions for the growth of rubber plants are high temperature (28 ± 2 ^o^C), high humidity, and rainfall of 2000–4000 mm/year. ^5^ Rubber plantations in marginal areas, such as the northeastern states of India, southern China, northern and northeastern Thailand, and eastern Indonesia, experience abiotic stresses such as drought. Indonesia has a wide drought area of about 122.1 million ha, and it is not optimally exploited due to limited water resources.

The response caused by drought is quite complex because it involves changes in morphology, physiology, and metabolism. The initial response to drought stress is loss of turgor pressure, which results in reduced growth rate, stem elongation, leaf senescence, and stomatal opening. Drought changes the source–sink relationship and affects the translocation of photosynthate to produce fruit quickly for certain crops. ^6^ The fastest response to a water deficit is the stomatal closure to protect plants from water shortages. Water deficit results in abscisic acid (ABA) biosynthesis, which triggers stomatal closure and causes a decrease in intracellular CO _2_ levels and the inhibition of photosynthesis. ^7^ Water shortages do not always promote these responses in all plant species. Lack of intracellular CO _2_ due to prolonged stomatal closure leads to the accumulation of reactive oxygen and nitrogen species, which damages the photosynthetic apparatus. ^8^ Besides that, the presence of osmoprotectants, such as proline, trehalose sugar, glycine betaine, D-onomitol, and mannitol maintain the growth and productivity of a plant experiencing drought stress. ^9^^–^^11^ The presence of antioxidant enzymes, such as superoxide dismutase (SOD), catalase (CAT), ascorbate peroxidase (APX), and glutathione reductase (GR), in cellular and cytoplasmic organelles plays an important role in the detoxification of these reactive oxygen species (ROS), and enables plant cells to activate various stress sensors, which will then activate various signal paths.

Inhibited growth is a typical symptom of drought stress. ^12^ The consequent physiological, biochemical, and molecular changes affect various cellular processes, thereby reducing the quantity and quality of the plant yield. In times of drought stress, lack of sufficient water combined with the increased CO _2_ in the atmosphere can cause plant death. ^13^ Based on bio-informatics, there are 20 proteins related to drought stress in rubber plants. ^14^ This study aims to determine the mechanism of drought-tolerant clones, based on physiological characteristics, to obtain character selection and drought-tolerant clones early.

## Methods

### Study area

Analysis of physiological characteristics was carried out at the Physiology and Protection Laboratory of the Unit Research Sungei Putih, Galang, Deli Serdang, North Sumatra. The study was carried out in a greenhouse during June 2020–May 2021. The materials used were red–yellow podzolic soil (water content = 3.9, pH = 4.5, C-organic = 0.92, N = 0.15, P _2_O _5_ = 2.13), compound fertilizer, Dithane M-45 and Triko-SP Plus. The tools used were polybags (18 × 35 cm and 50×60 cm), a hoe, soil sieve, bucket, watering can, 100 kg UK scale, analytical balance, object glass, deck glass, binocular microscope, water bath, vortex, UV spectrophotometer, filter paper, test tube, gloves, mask, tissue, distilled water, mortar, beaker, micropipettes 1 ml and 100 μl, stirrer, 15 watt lamp, microcentrifuge, microwave, and others.

### Experimental design

The study was arranged based on a factorial randomized block design (RBD). The first factor was the type of clone, consisting of seven types, namely C1: IRR 425, C2: IRR 428, C3: IRR 429, C4: IRR 434, C5: IRR 440, C6: RRIC 100, and C7: BPM 24. The second factor was water content, consisting of three levels, namely: W1: 30%, W2: 60%, and W3: 90%. Each experimental unit was repeated three times, and as many as 63 samples were observed.

### Data analysis

Observations were carries out six times on physiological characteristics, with time intervals every three weeks. If the test of variance obtained significantly different treatments, then the Tukey distance test of 0.5% was carried out. ^15^ The characteristics observed were total sugar content, ^16^ chlorophyll a, b, total, ^17^ proline, ^18^^–^^21^ super peroxidase dismutase (SOD), ^22^ peroxidase dismutase (POD), ^23^ APX enzyme, ^24^ and hydrogen peroxide (H _2_O _2_). ^22^

A step-by-step description of the procedure to analyze sugar content, proline, chlorophyll a, b, total, SOD, POD, H2O2 and APX has been deposited in prototocol.io and is available at dx.doi.org/10.17504/protocols.io.5jyl8je1dg2w/v1.

## Results

### Total sugar content (μM)

The total sugar content analysis showed a significant effect in all the observations except the first one ( Table 1).

**Table 1.** The total sugar content (μM) in the six observations of rubber clones.

Observation	Level	Total sugar content (μM)	Clone
1 ^st^	Highest	195.05	RRIC 100
	Lowest	158.99	IRR 425
2 ^nd^	Highest	129.34	RRIC 100
	Lowest	65.53	IRR 425
3 ^rd^	Highest	114.9	RRIC 100
	Lowest	73.28	BPM 24
4 ^th^	Highest	158.75	BPM 24
	Lowest	105.17	IRR 425
5 ^th^	Highest	221.09	IRR 429
	Lowest	183.01	IRR 440
6 ^th^	Highest	184.73	RRIC 100
	Lowest	136.85	IRR 425

The total sugar content in the six observations carried out on tested clones were consistent. The RRIC 100 clone had the highest total sugar content four times, and the IRR 425 clone had the lowest three times.

The total sugar content analysis at different water levels generally showed a significant effect, except for the initial observation. This indicates that, in most of the six observations, water content affects the total sugar content of the tested clones ( Table 2).

**Table 2.** Total sugar content (μM) at different water content levels (%).

Observation	Level	Total sugar content (μM)	Water content (%)
1 ^st^	Highest	188.24	30
	Lowest	172.51	90
2 ^nd^	Highest	111.68	30
	Lowest	85.03	90
3 ^rd^	Highest	116.79	30
	Lowest	77.69	90
4 ^th^	Highest	151.64	30
	Lowest	112.89	90
5 ^th^	Highest	200.57	30
	Lowest	205.76	90
6 ^th^	Highest	178.83	30
	Lowest	142.74	90

Analysis of the total sugar content due to the interaction between the type of clone and water content level (30%, 60%, 90%) showed significant differences, except in the first observation ( Table 3).

**Table 3.** Total sugar content (μM) due to interactions between clone type and water content (%).

Observation	Level	Total sugar content (μM)	Clone	Water content (%)
1 ^st^	Highest	237.00	IRR 428	90
	Lowest	137.90	IRR 425	90
2 ^nd^	Highest	149.85	IRR 429	30
	Lowest	61.03	IRR 428	90
3 ^rd^	Highest	151.18	RRIC 100	30
	Lowest	65.70	BPM 24	60
4 ^th^	Highest	206.06	IRR 429	30
	Lowest	94.52	IRR 425	60
5 ^th^	Highest	227.64	IRR 429	30
	Lowest	152.74	RRIC 100	60
6 ^th^	Highest	215.48	RRIC 100	30
	Lowest	121.48	IRR 429	90

What is interesting about these results is that the highest accumulation of total sugar is seen in the application of 30% water content. Meanwhile the effect on the different types of clones was quite diverse. The IRR 429 had the highest total sugar in three observations (second, fourth and fifth). The RRIC 100 had the highest total sugar in two observations (third and fourth). The two clones, RRIC 100 and IRR 429, also had the lowest total sugar in the fifth and sixth observations, respectively. The complete dataset of total sugar content is displayed in Supplementary Table 1.

**Figure 1.**
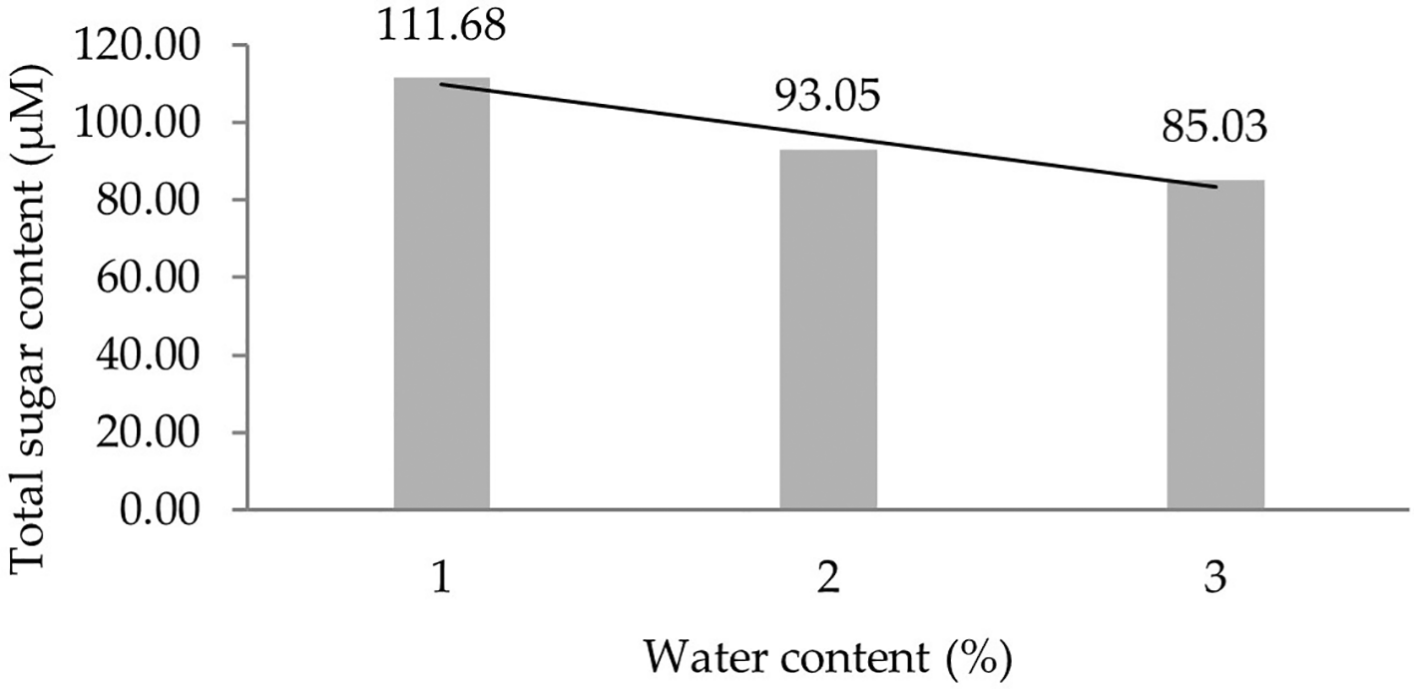
Pattern of total sugar content linear curve as a result of orthogonal polynomial analysis. 1: 30%; 2: 60%; 3: 90%.

Two forms of polynomial curves can be the effect of water content and can be shown by the orthogonal polynomial regression obtained from three levels of water content, namely linear and cubic curves. The results of the analysis show that the linear curve shows a real effect. Figure 1 shows the linear curve regression pattern formed in detail. It demonstrates that the lower the water content added to the growing media, the higher the total sugar content derived from the leaf analysis of several rubber clones of IRR 400 series, RRIC 100, and BPM 24.

### Proline (mgg ^-1^)

Table 4 depicts the proline analysis of clone types treated with different water contents and shows that there were significantly different effects in all observations.

**Table 4.** Proline levels (mgg ^-1^) in different clones.

Observation	Level	Proline (mgg ^-1^)	Clones
1 ^st^	Highest	11.16	IRR 429
	Lowest	5.89	RRIC 100
2 ^nd^	Highest	9.27	IRR 429
	Lowest	5.74	IRR 428
3 ^rd^	Highest	13.66	BPM 24
	Lowest	8.76	IRR 440
4 ^th^	Highest	8.11	IRR 425
	Lowest	6.25	IRR 440
5 ^th^	Highest	8.95	IRR 425
	Lowest	5.33	IRR 440
6 ^th^	Highest	6.86	IRR 425
	Lowest	5.69	IRR 440

The results of proline analysis at different water content percentages showed significantly different effects in all observations, as shown in Table 5.

**Table 5.** Proline levels (mgg ^-1^) at different water content levels (%).

Observation	Level	Proline (mgg ^-1^)	Water content (%)
1 ^st^	Highest	10.94	30
	Lowest	5.89	90
2 ^nd^	Highest	9.31	30
	Lowest	5.12	90
3 ^rd^	Highest	11.74	30
	Lowest	9.96	90
4 ^th^	Highest	7.91	30
	Lowest	6.65	60
5 ^th^	Highest	7.72	30
	Lowest	6.28	90
6 ^th^	Highest	6.28	30
	Lowest	5.78	60

The proline analysis caused by the interaction between rubber clones IRR 400 series, RRIC 100, and BPM 24 and given water content (30%, 60%, 90%) showed significantly different effects in all observations, as displayed in Table 6.

**Table 6.** Proline levels (mgg ^-1^) due to interactions between clone type and water content (%).

Observation	Level	Proline (mgg ^-1^)	Clone	Water content (%)
1 ^st^	Highest	13.80	IRR 425	30
	Lowest	1.42	RRIC 100	90
2 ^nd^	Highest	14.87	IRR 429	30
	Lowest	1.32	RRIC 100	90
3 ^rd^	Highest	17.61	IRR 434	30
	Lowest	5.35	IRR 440	90
4 ^th^	Highest	9.43	IRR 425	30
	Lowest	5.14	RRIC 100	90
5 ^th^	Highest	12.32	IRR 425	30
	Lowest	2.42	IRR 440	90
6 ^th^	Highest	9.13	IRR 428	30
	Lowest	4.41	BPM 24	90

**Figure 2.**
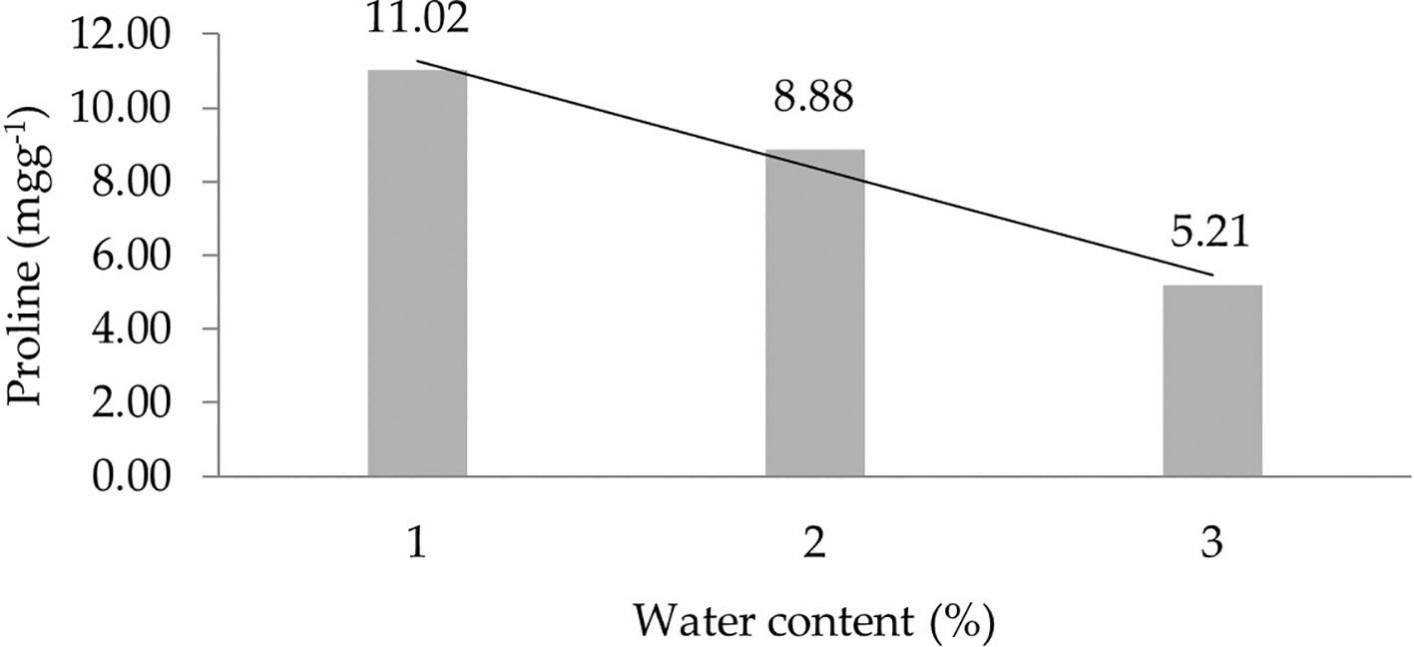
Pattern of proline linear curve as a result of orthogonal polynomial analysis. 1: 30%; 2: 60%; 3: 90%.

The assessment of orthogonal polynomial regression showed a linear curve, where the water content at the 30% level had the highest proline value. The orthogonal polynomial linear curve pattern of proline characteristics of several rubbers of IRR 400 series, RRIC 100, and BPM 24 is shown in Figure 2. The complete dataset of proline can be seen in Supplementary Table 2.

### Chlorophyll a (μgmg ^-1^)

The chlorophyll a analysis on the different clone types showed a significant effect, except for the first observation, as displayed in Table 7.

**Table 7.** Chlorophyll a levels (μgmg ^-1^) in different clones.

Observation	Level	Chlorophyll a (μgmg ^-1^)	Clones
1 ^st^	Highest	0.25	IRR 425
	Lowest	0.22	BPM 24
2 ^nd^	Highest	0.60	IRR 425
	Lowest	0.46	BPM 24
3 ^rd^	Highest	0.46	IRR 425
	Lowest	0.41	BPM 24
4 ^th^	Highest	0.60	IRR 425
	Lowest	0.49	BPM 24
5 ^th^	Highest	0.62	IRR 425
	Lowest	0.45	BPM 24
6 ^th^	Highest	0.72	IRR 425
	Lowest	0.52	BPM 24

Table 8 shows the chlorophyll a analysis at different water contents, which demonstrated a significant effect in all six observations.

**Table 8.** Chlorophyll a levels (μgmg ^-1^) at different water content levels (%).

Observation	Level	Chlorophyll a (μgmg ^-1^)	Water content (%)
1 ^st^	Highest	0.28	30
	Lowest	0.20	90
2 ^nd^	Highest	0.64	30
	Lowest	0.40	90
3 ^rd^	Highest	0.54	30
	Lowest	0.33	90
4 ^th^	Highest	0.67	30
	Lowest	0.43	90
5 ^th^	Highest	0.57	30
	Lowest	0.36	90
6 ^th^	Highest	0.59	30
	Lowest	0.39	90

Analysis of chlorophyll a levels due to the interaction between clones and water content (30%, 60%, 90%) showed significant differences in all six observations ( Table 9). The complete dataset of chlorophyll a is depicted in Supplementary Table 3.

**Table 9.** Chlorophyll a levels (μgmg ^-1^) due to interactions between clones and water content (%).

Observation	Level	Chlorophyll a (μgmg- ^1^)	Clones	Water content (%)
1 ^st^	Highest	0.3	IRR 425	30
	Lowest	0.1	IRR 425	90
2 ^nd^	Highest	0.8	IRR 429	30
	Lowest	0.3	IRR 440	90
3 ^rd^	Highest	0.7	IRR 425	30
	Lowest	0.3	IRR 425	90
4 ^th^	Highest	0.9	RRIC 100	30
	Lowest	0.3	IRR 440	90
5 ^th^	Highest	0.8	IRR 425	30
	Lowest	0.2	IRR 429	90
6 ^th^	Highest	0.9	IRR 425	30
	Lowest	0.3	IRR 429	90

### Chlorophyll b (μgmg ^-1^)

The results of the chlorophyll b analysis with different clone types showed significantly different results, except for the first observation ( Table 10).

**Table 10.** Chlorophyll b levels (μgmg ^-1^) in different clones.

Observation	Level	Chlorophyll b (μgmg ^-1^)	Clones
1 ^st^	Highest	0.24	IRR 425
	Lowest	0.20	BPM 24
2 ^nd^	Highest	0.57	IRR 425
	Lowest	0.30	IRR 434
3 ^rd^	Highest	0.45	RRIC 100
	Lowest	0.33	IRR 434
4 ^th^	Highest	0.57	IRR 425
	Lowest	0.34	IRR 434
5 ^th^	Highest	0.56	BPM 24
	Lowest	0.46	IRR 429
6 ^th^	Highest	0.56	IRR 440
	Lowest	0.37	IRR 429

The results of analysis of chlorophyll b levels at the given water contents showed a significant effect in all six observations ( Table 11).

**Table 11.** Chlorophyll b levels (μgmg ^-1^) at different water content levels (%).

Observation	Level	Chlorophyll b (μgmg ^-1^)	Water content (%)
1 ^st^	Highest	0.25	30%
	Lowest	0.20	90%
2 ^nd^	Highest	0.52	30%
	Lowest	0.38	90%
3 ^rd^	Highest	0.49	30%
	Lowest	0.35	90%
4 ^th^	Highest	0.60	30%
	Lowest	0.41	90%
5 ^th^	Highest	0.61	30%
	Lowest	0.39	90%
6 ^th^	Highest	0.59	30%
	Lowest	0.38	90%

The analysis of chlorophyll b levels due to the interaction between clones and water content (30%, 60%, 90%) showed significant differences in all six observations, as shown in Table 12.

**Table 12.** Chlorophyll b levels (μgmg ^-1^) due to interactions between clones and water content (%).

Observation	Level	Chlorophyll b (μgmg ^-1^)	Clone	Water content (%)
1 ^st^	Highest	0.3	IRR 425	30
	Lowest	0.2	IRR 428	90
2 ^nd^	Highest	0.7	IRR 425	30
	Lowest	0.3	IRR 434	90
3 ^rd^	Highest	0.6	BPM 24	30
	Lowest	0.3	IRR 434	90
4 ^th^	Highest	0.7	RRIC 100	30
	Lowest	0.3	IRR 434	90
5 ^th^	Highest	0.7	BPM 24	30
	Lowest	0.3	IRR 440	90
6 ^th^	Highest	0.7	IRR 425	30
	Lowest	0.3	IRR 429	90

**Figure 3.**
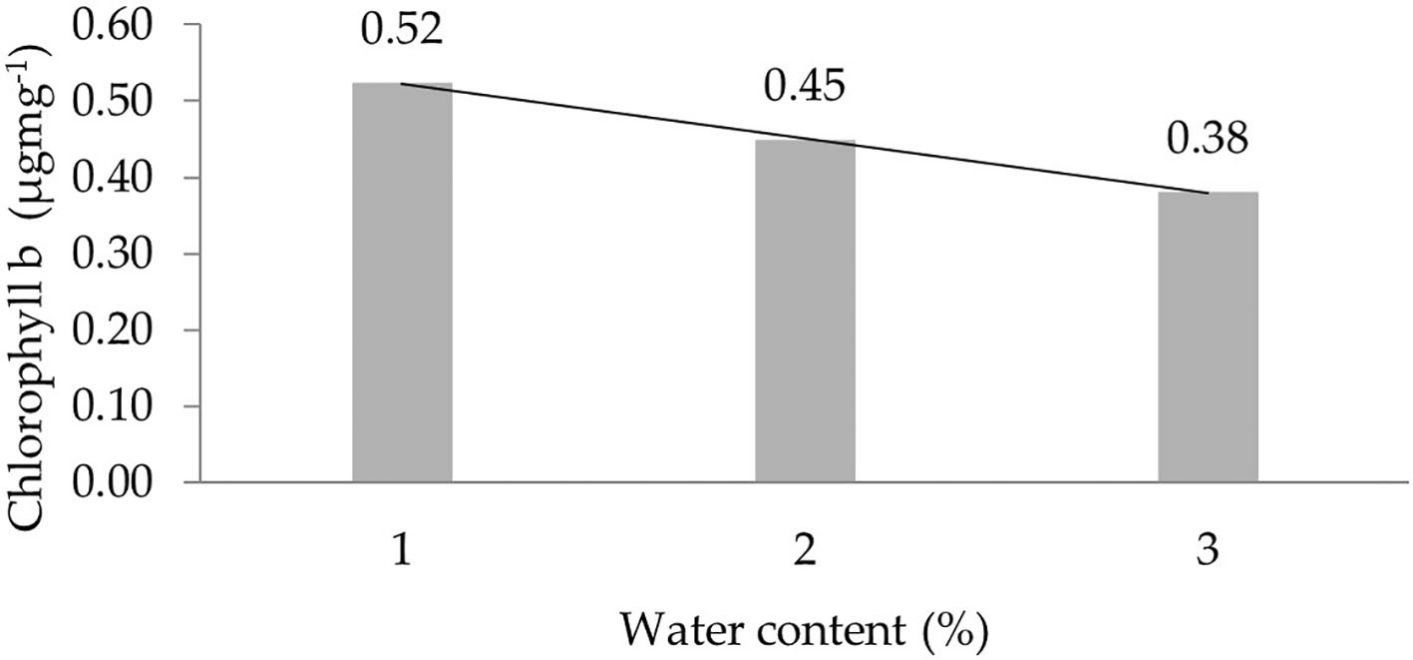
Pattern of chlorophyll b linear curve as a result of orthogonal polynomial analysis. 1: 30%; 2: 60%; 3: 90%.

The assessment of the orthogonal polynomial regression showed a linear curve, where the water content at the 30% level had the highest chlorophyll b value. The orthogonal polynomial linear curve pattern of the chlorophyll b characteristics of several rubber clones of IRR 400 series, RRIC 100, and BPM 24 can be seen in Figure 3. The complete dataset of chlorophyll b can be seen in Supplementary Table 4.

### Chlorophyll total (μgmg ^-1^)

The analysis results of chlorophyll total with different clone types showed significantly different results except for one observation ( Table 13).

**Table 13.** Chlorophyll total levels (μgmg ^-1^) in different clones.

Observation	Level	Chlorophyll total (μgmg ^-1^)	Clone
1 ^st^	Highest	0.48	IRR 425
	Lowest	0.42	BPM 24
2 ^nd^	Highest	1.17	IRR 425
	Lowest	0.77	IRR 434
3 ^rd^	Highest	1.00	RRIC 100
	Lowest	0.66	IRR 434
4 ^th^	Highest	1.28	RRIC 100
	Lowest	0.78	IRR 440
5 ^th^	Highest	1.17	IRR 425
	Lowest	0.74	IRR 440
6 ^th^	Highest	1.24	IRR 425
	Lowest	0.74	IRR 429

The results of chlorophyll total analysis with the given water content showed a significant effect in all six observations, as depicted in Table 14.

**Table 14.** Chlorophyll total levels (μgmg ^-1^) at different water content levels (%).

Observation	Level	Chlorophyll total (μgmg ^-1^)	Water content (%)
1 ^st^	Highest	0.53	30
	Lowest	0.40	90
2 ^nd^	Highest	1.16	30
	Lowest	0.78	90
3 ^rd^	Highest	1.03	30
	Lowest	0.69	90
4 ^th^	Highest	1.27	30
	Lowest	0.83	90
5 ^th^	Highest	1.19	30
	Lowest	0.75	90
6 ^th^	Highest	1.18	30
	Lowest	0.77	90

The analysis of chlorophyll total levels due to the interaction between IRR 400 series, RRIC 100, and BPM 24 and water content (30%, 60%, 90%) showed significant differences in all six observations ( Table 15). The complete dataset of chlorophyll total can be seen in Supplementary Table 5.

**Table 15.** Chlorophyll total levels (μgmg ^-1^) due to interactions between clones and water content (%).

Observation	Level	Chlorophyll total (μgmg ^-1^)	Clone	Water content (%)
1 ^st^	Highest	0.6	IRR 429	30
	Lowest	0.3	IRR 425	90
2 ^nd^	Highest	1.4	IRR 425	30
	Lowest	0.7	IRR 434	90
3 ^rd^	Highest	1.2	IRR 425	30
	Lowest	0.6	IRR 425	90
4 ^th^	Highest	1.7	RRIC 100	30
	Lowest	0.7	IRR 440	90
5 ^th^	Highest	1.4	IRR 425	30
	Lowest	0.6	IRR 440	90
6 ^th^	Highest	1.6	IRR 425	30
	Lowest	0.6	IRR 429	90

**Figure 4.**
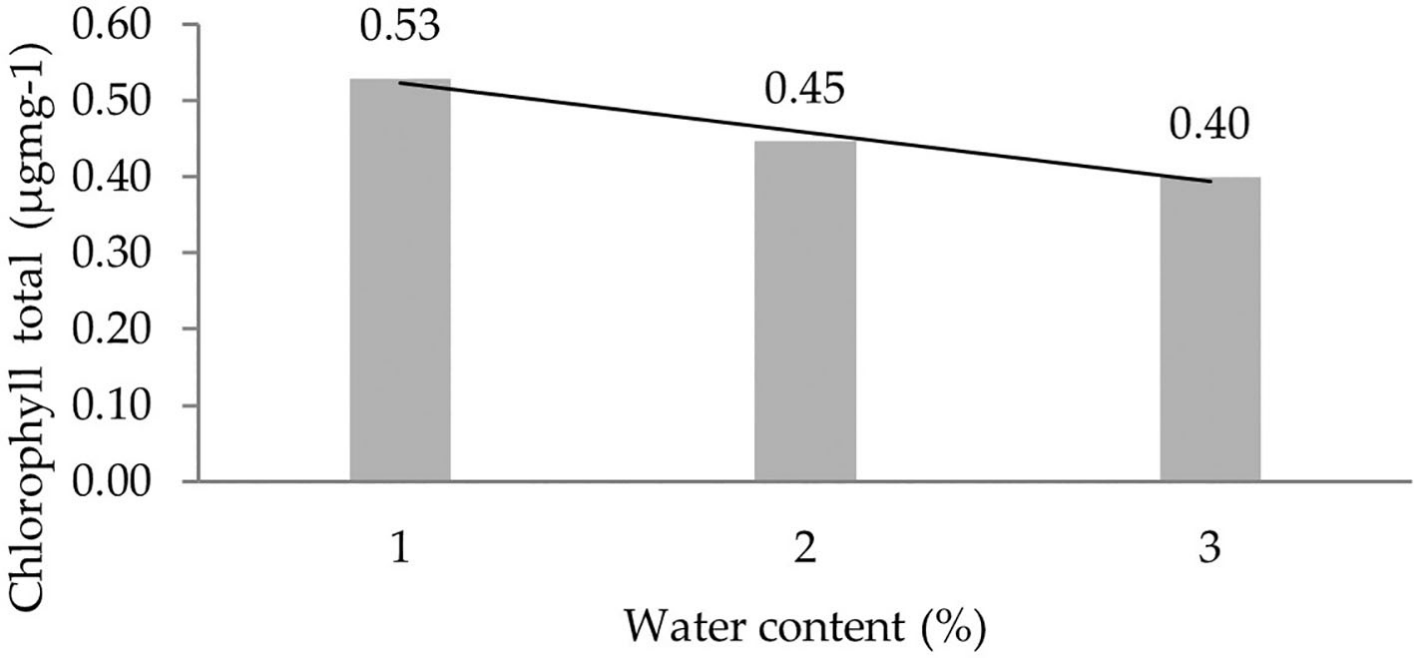
Pattern of chlorophyll total linear curve as a result of orthogonal polynomial analysis. 1: 30%; 2: 60%; 3: 90%.

Orthogonal polynomial regression shows a linear curve, where the water content at 30% has the highest total chlorophyll value. The linear curve shows that the total chlorophyll content increases with the decreasing water content. The orthogonal polynomial linear curve pattern of chlorophyll total of several clones of IRR 400 series, RRIC 100, and BPM 24 can be seen in Figure 4.

### Hydrogen peroxidase/H _2_O _2_ (μmolg ^-1^)

The results of the H _2_O _2_ analysis with different types of clones showed a significantly different effect in two of the observations (third and fourth) ( Table 16).

**Table 16.** H _2_O _2_ levels (μmolg ^-1^) in different clones.

Observation	Level	H _2_O _2_ (μmolg ^-1^)	Clone
1 ^st^	Highest	0.80	IRR 428
	Lowest	0.77	IRR 429
2 ^nd^	Highest	0.81	IRR 440
	Lowest	0.75	IRR 425
3 ^rd^	Highest	0.79	RRIC 100
	Lowest	0.74	IRR 429
4 ^th^	Highest	0.79	IRR 440
	Lowest	0.73	IRR 428
5 ^th^	Highest	0.75	RRIC 100
	Lowest	0.74	IRR 425
6 ^th^	Highest	0.74	IRR 425
	Lowest	0.72	IRR 434

The results of H _2_O _2_ analysis at different water content levels did not show significant differences in any of the observation ( Table 17).

**Table 17.** H _2_O _2_ levels (μmolg ^-1^) at different water content levels (%).

Observation	Level	H _2_O _2_ (μmolg ^-1^)	Water content (%)
1 ^st^	Highest	0.79	30
	Lowest	0.78	60
2 ^nd^	Highest	0.78	30
	Lowest	0.76	90
3 ^rd^	Highest	0.77	60
	Lowest	0.76	90
4 ^th^	Highest	0.77	90
	Lowest	0.75	30
5 ^th^	Highest	0.75	60
	Lowest	0.74	30
6 ^th^	Highest	0.73	90
	Lowest	0.72	30

The analysis of H _2_O _2_ levels (μmolg ^-1^) due to interactions between IRR 400 series, RRIC 100, and BPM 24 and given water content (30%, 60%, 90%) showed a significantly different effect in just one observation (fourth) ( Table 18). The complete dataset of H _2_O _2_ is displayed in Supplementary Table 6.

**Table 18.** H _2_O _2_ levels (μmolg ^-1^) due to interactions between clones and water content (%).

Observation	Level	H _2_O _2_ (μmolg ^-1^)	Clone	Water content (%)
1 ^st^	Highest	0.8	IRR 428	30
	Lowest	0.7	IRR 429	60
2 ^nd^	Highest	0.8	IRR 428	30
	Lowest	0.7	IRR 429	60
3 ^rd^	Highest	0.8	BPM 24	60
	Lowest	0.7	IRR 429	60
4 ^th^	Highest	0.8	BPM 24	90
	Lowest	0.7	IRR 429	30
5 ^th^	Highest	0.8	BPM 24	60
	Lowest	0.7	IRR 425	30
6 ^th^	Highest	0.8	IRR 425	90
	Lowest	0.7	IRR 434	60

**Figure 5.**
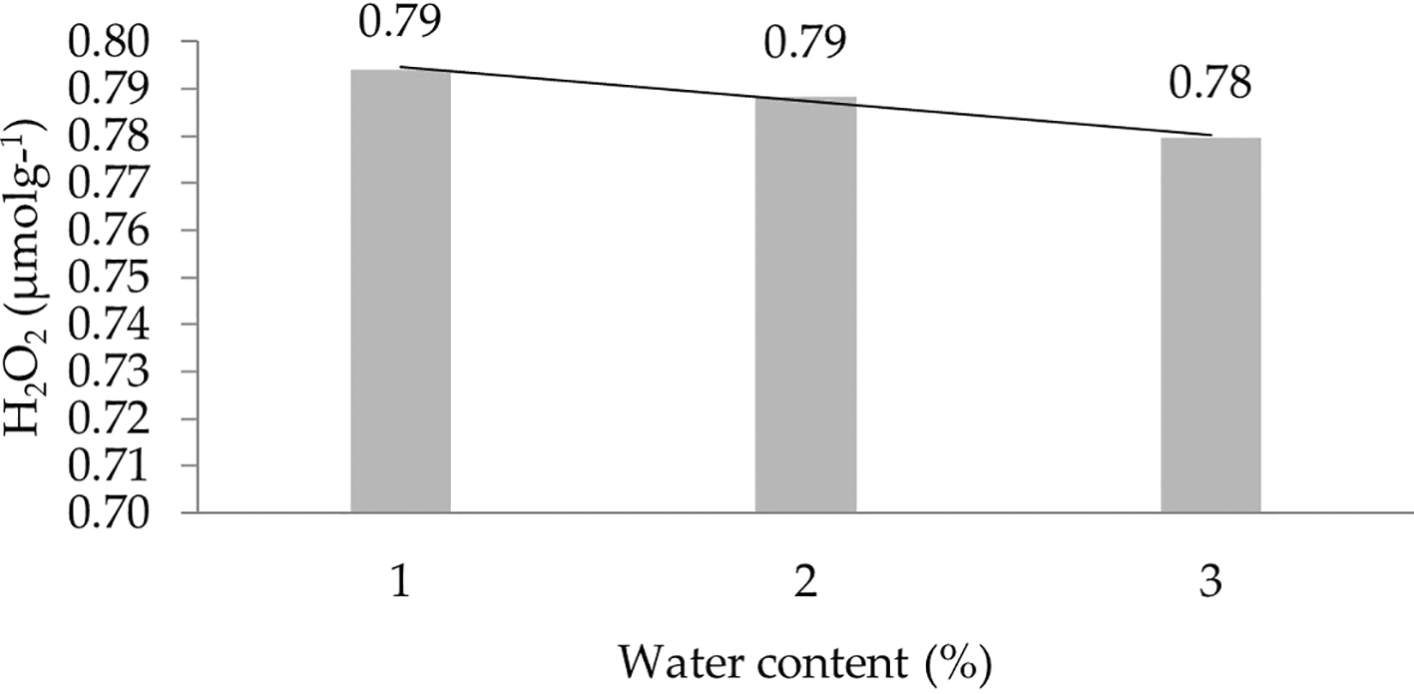
Pattern of H _2_O _2_ (μmolg ^-1^) linear curve as a result of orthogonal polynomial analysis. 1: 30%; 2: 60%; 3: 90%.

The effect of water content on the H _2_O _2_ characteristic shows a linear regression curve based on orthogonal polynomials. This indicates that the lower the water content, the higher the concentration of H _2_O _2_. The linear regression pattern between H _2_O _2_ content and water content can be seen in Figure 5.

### Ascorbate peroxidase/APX (unitsmg ^-1^)

The results of APX analysis with different clone types were not significantly different in any of the observations ( Table 19).

**Table 19.** APX levels (unitsmg ^-1^) in different clones.

Observation	Level	APX (unitsmg ^-1^)	Clones
1 ^st^	Highest	1.48	IRR 425
	Lowest	1.22	RRIC 100
2 ^nd^	Highest	1.32	IRR 429
	Lowest	1.13	IRR 425
3 ^rd^	Highest	1.10	RRIC 100
	Lowest	1.00	BPM 24
4 ^th^	Highest	1.44	IRR 434
	Lowest	1.21	IRR 425
5 ^th^	Highest	1.41	IRR 428
	Lowest	1.24	BPM 24
6 ^th^	Highest	1.14	IRR 425
	Lowest	0.96	IRR 434

The analysis results of APX at different water content levels were not significantly different in any of the observations ( Table 20).

**Table 20.** APX levels (unitsmg ^-1^) at different water content levels (%).

Observation	Level	APX (unitmg ^-1^)	Water content (%)
1 ^st^	Highest	1.38	30
	Lowest	1.24	90
2 ^nd^	Highest	1.34	60
	Lowest	1.15	30
3 ^rd^	Highest	1.07	60
	Lowest	1.05	90
4 ^th^	Highest	1.35	60
	Lowest	1.33	90
5 ^th^	Highest	1.38	60
	Lowest	1.33	90
6 ^th^	Highest	1.09	90
	Lowest	1.05	30

The analysis of APX levels (unitmg ^-1^) due to the interaction between IRR 400 series, RRIC 100, and BPM 24 and water content (30%, 60%, 90%) did not show any significant differences in any of the observations ( Table 21). The complete dataset of APX can be seen in Supplementary Table 7.

**Table 21.** The APX levels (unitmg ^-1^) due to interactions between clones and water content (%).

Observation	Level	APX (unitmg ^-1^)	Clones	Water content (%)
1 ^st^	Highest	0.8	IRR 428	30
	Lowest	0.7	IRR 429	60
2 ^nd^	Highest	0.8	IRR 428	30
	Lowest	0.7	IRR 429	60
3 ^rd^	Highest	0.8	BPM 24	60
	Lowest	0.7	IRR 429	60
4 ^th^	Highest	0.8	BPM 24	90
	Lowest	0.7	IRR 429	30
5 ^th^	Highest	0.8	BPM 24	60
	Lowest	0.7	IRR 425	30
6 ^th^	Highest	0.7	IRR 425	90
	Lowest	0.3	IRR 434	60

### Superoxide Dismutase/SOD (unitmg ^-1^)

The SOD analysis with clone types showed a significant difference in three of the observations ( Table 22).

**Table 22.** SOD levels (unitmg ^-1^) in different clones.

Observation	Level	SOD (unitmg ^-1^)	Clones
1 ^st^	Highest	2.43	IRR 425
	Lowest	2.23	IRR 429
2 ^nd^	Highest	2.70	IRR 440
	Lowest	2.44	RRIC 100
3 ^rd^	Highest	2.44	IRR 440
	Lowest	2.21	IRR 429
4 ^th^	Highest	2.35	IRR 425
	Lowest	2.23	IRR 434
5 ^th^	Highest	2.44	IRR 429
	Lowest	2.33	RRIC 100
6 ^th^	Highest	2.62	RRIC 100
	Lowest	2.40	IRR 440

Analysis of SOD (unitmg ^-1^) levels at different water content levels showed significant differences in three of the observations, as depicted in Table 23.

**Table 23.** SOD levels (unitmg ^-1^) at different water content levels (%).

Observation	Level	SOD (unitmg ^-1^)	Water content (%)
1 ^st^	Highest	2.42	60
	Lowest	2.30	90
2 ^nd^	Highest	2.59	90
	Lowest	2.53	60
3 ^rd^	Highest	2.27	90
	Lowest	2.23	30
4 ^th^	Highest	2.32	30
	Lowest	2.28	60
5 ^th^	Highest	2.45	60
	Lowest	2.36	90
6 ^th^	Highest	2.52	90
	Lowest	2.43	60

The analysis of SOD levels due to interaction between IRR 400 series, RRIC 100, and BPM 24 and water content (30%, 60%, 90%) showed significant differences in two observations ( Table 24). The complete dataset of SOD can be seen in Supplementary Table 8.

**Table 24.** SOD levels (unitmg ^-1^) due to interactions of clones and water content (%).

Observation	Level	SOD (unitmg ^-1^)	Clone	Water content (%)
1 ^st^	Highest	2.6	IRR 425	30
	Lowest	2.2	IRR 429	90
2 ^nd^	Highest	2.7	IRR 440	90
	Lowest	2.4	RRIC 100	90
3 ^rd^	Highest	2.4	IRR 440	30
	Lowest	2.1	BPM 24	90
4 ^th^	Highest	2.5	IRR 425	30
	Lowest	2.2	IRR 425	60
5 ^th^	Highest	2.6	IRR 428	60
	Lowest	2.3	IRR 440	90
6 ^th^	Highest	2.7	BPM 24	30
	Lowest	2.3	IRR 440	30

### Peroxide dismutase/POD (unitsmg ^-1^)

The POD analysis with different types of clones showed significant differences in two observations, as shown in Table 25.

**Table 25.** POD levels (unitmg ^-1^) in different clones.

Observation	Level	POD (unitmg ^-1^)	Clone
1 ^st^	Highest	0.90	IRR 428
	Lowest	0.87	IRR 425
2 ^nd^	Highest	0.94	IRR 428
	Lowest	0.89	IRR 429
3 ^rd^	Highest	0.90	RRIC 100
	Lowest	0.85	IRR 428
4 ^th^	Highest	0.94	BPM 24
	Lowest	0.85	IRR 434
5 ^th^	Highest	0.92	RRIC 100
	Lowest	0.87	IRR 429
6 ^th^	Highest	0.88	BPM 24
	Lowest	0.83	IRR 429

The analysis of POD levels at different water content levels showed a significant difference in one observation, as depicted in Table 26.

**Table 26.** POD levels (unitmg ^-1^) at different water content levels (%).

Observation	Level	POD (unitmg ^-1^)	Water content (%)
1 ^st^	Highest	0.890	60
	Lowest	0.870	30
2 ^nd^	Highest	0.920	60
	Lowest	0.900	90
3 ^rd^	Highest	0.875	30
	Lowest	0.874	90
4 ^th^	Highest	0.914	90
	Lowest	0.906	60
5 ^th^	Highest	0.920	30
	Lowest	0.910	60
6 ^th^	Highest	0.860	30
	Lowest	0.830	90

The analysis of POD levels due to interaction between IRR 400 series, RRIC 100, and BPM 24 and given water content (30%, 60%, 90%) showed a significant difference in just one observation ( Table 27). The complete dataset of POD can be seen in Supplementary Table 9.

**Table 27.** POD levels (unitmg ^-1^) due to interaction of clones and water content (%).

Observation	Level	POD (unitmg ^-1^)	Clones	Water content (%)
1 ^st^	Highest	1.0	IRR 440	90
	Lowest	0.8	IRR 428	30
2 ^nd^	Highest	1.0	IRR 428	90
	Lowest	0.9	RRIC 100	90
3 ^rd^	Highest	0.9	IRR 434	90
	Lowest	0.8	IRR 434	30
4 ^th^	Highest	1.0	BPM 24	30
	Lowest	0.8	IRR 434	30
5 ^th^	Highest	1.0	IRR 434	90
	Lowest	0.9	IRR 434	90
6 ^th^	Highest	0.9	BPM 24	30
	Lowest	0.8	IRR 434	90

## Discussion

Physiological characteristics that arise due to plant activities in certain environments are observable and enable growth and development. The accumulation of osmoprotectants is a key biochemical property in plants tolerant to abiotic stress, ^10^^,^^21^ and there is clear evidence that osmotic adjustment sustains crop yields under drought stress. ^9^ Drought stress causes changes in amino acid metabolism. The accumulated solutes protect cellular proteins, organelles, membranes and various enzymes against drought stress.

Several physiological characteristics were analyzed to see the effect of water content on IRR 400 series, RRIC 100, and BPM 24 rubber clones. Some of the dissolved substances assessed in this study were total sugar, proline, and chlorophyll (a, b, total). The correlation of total sugar content to each clone showed different effects. Each clone showed its ability to produce total sugar content when stressed. The RRIC 100 is a dry tolerant clone in the field. The increase in total sugar content was seen in most of observations of water content treatment. The interaction between clone type and water content can increase total sugar content, especially when the water content added is 30%. Initial hypotheses suggest that each clone has the ability to adapt to water shortages. The accumulation of soluble sugars in plant cells subjected to drought stress is responsible for the osmotic adjustment. ^25^ Sugar accumulation in drought-stressed plants is controlled by several mechanisms that affect soluble sugar formation and transfer in leaves. ^26^ Similar results of increased total sugar accumulation have been produced in drought-stressed soybeans ^26^ and sugarcane. ^27^

This study showed different proline values among the tested clones. All six observations indicate that clones have the ability to survive drought. The IRR 425 clone had the highest proline levels in four observations. Meanwhile, the IRR 440 had the lowest proline levels in four observations. Assessing by the proline characteristic, the initial assumption was that IRR 425 had a stronger adaptation compared with other clones, especially the IRR 440. Regarding different water contents (30%, 60%, 90%) the proline levels at 30% were greater than at 60% and 90%. This indicated that a higher amount of proline accumulated when the water content was lower in the growth medium. Proline is an important amino acid as it is an osmotic compatible molecule and has the potential to form a defense system to increase drought tolerance. Proline acts as antioxidative defense molecule and causes stress signaling. ^12^ It is classified as an osmoprotectant, which causes increased hyperosmolarity and increased activity of antioxidant enzymes. ^28^ Increased proline content in drought-stress plants can provide high energy to increase plant growth in water-deficit conditions. ^29^ Hence, proline accumulation correlates with osmoprotection. ^30^ The interaction between clone types and moisture content indicated that each clone showed a different effect in the six observations. The clones had high proline levels when treated with 30% water content. This shows that the clonal factor still has to be tested in other environments against drought stress. The proline content has been shown to increase about 10-fold in mungbean, ^31^ maize, ^32^ millet, ^12^^,^^33^^,^^34^ nyamplung, ^35^ and soybean ^26^ under drought stress.

Chlorophyll is the main pigment found in chloroplasts. ^36^ The three main functions of chlorophyll in the photosynthesis process are harnessing solar energy, triggering CO _2_ fixation to produce carbohydrates, and providing energy for the ecosystem as a whole. Chlorophyll a and chlorophyll b absorb the most light in the red part (600–700 nm), and absorb the least in the green part (500–600 nm). ^35^^–^^37^ In this study, it was seen that chlorophyll a, b, and total levels at 30% were higher than 90%. This is presumably because the rubber plant is an annual plant that is able to adapt to water shortages as its root structure, taproot, grows deeper to find water further from the soil surface. In addition, when stressed, the lateral roots will grow more to take advantage of the surface water. Even though the plants are grown in greenhouses, the water supplied into the planting media will not go down because the planting media is designed to not have holes, gaps, or place for water to come out. In addition, the surface of the polybag is also covered by plastic to minimize the occurrence of evapotranspiration from the growing media.

Antioxidants are active substances that naturally detoxify free radicals (ROS). The presence of oxidative stress and an abundance of antioxidants are important activities for metabolic protection when plants are under stress. ROS in the form of free radicals and peroxides are molecules derived from oxygen metabolism. The toxic effects of ROS can be countered by antioxidant enzymatic as well as non-enzymatic systems, such as SOD, CAT, APX, GR, ascorbic acid (AsA), tocopherols, glutathione and phenolic compounds, and others. Typically, each cellular compartment contains more than one enzymatic activity that detoxifies an ROS. The presence of these enzymes in almost all cells plays an important role in ROS detoxification for plant survival. ^38^

H _2_O _2_ has several important roles in various biochemical and physiological processes. Long plant life and long growth processes result in H _2_O _2_ crossing cellular membranes and potentially acting as a signal in the signal transduction pathway of stress. This pathway triggers various responses of the adaptation process in the environment where the plant is cultivated. ^39^ High levels of H _2_O _2_ cause oxidative stress, which then causes cell damage and death. ^40^ However, optimal levels of H _2_O _2_ can increase tolerance to abiotic stresses through modulation of various physiological processes, including photosynthesis, opening and closing of stomata, osmotic adjustment, and ROS detoxification. ^39^^,^^40^ ROS detoxification is very important in maintaining the structural and membrane integrity of cellular organelles and keeping them fully functional under stress. The accumulation of optimal amounts of H _2_O _2_ triggers the occurrence of chitinase proteins that can produce calcium homeostasis, ion channels, phosphatases, transcription factors, and abscisic acid (ABA), signaling responses to stress. ^41^

APX in ascorbate–glutathione (AsA–GSH) cycling enzymes is responsible for the decomposition of H _2_O _2_ produced by SOD in different cellular organelles. APX plays a key role in both drought stress response and recovery after drought. ^41^^,^^42^ APX is an integral component of the (ASC–GSH) cycle. APX performs the same function in the cytosol and chloroplasts. APX reduces H _2_O _2_ to H _2_O and docosahexaenoic acid (DHA), using AsA as a reducing agent.

$$H_{2}O_{2}+\mathrm{AA}\to {\;}_{2}H_{2}O+\mathrm{DHA}$$

The APX family consists of five isoforms based on different sites of amino acid formation, such as the cytosol, mitochondria, peroxisomes, and chloroplastids (stroma and thylakoids). ^43^ APX is widely distributed and has a better affinity to H _2_O _2_, especially in terms of more efficient uptake of H _2_O _2_ in times of stress. ^43^^–^^46^

Thought the SOD levels in each clone showed a significant effect due to water content, it was limited to a few observations because drought affects the metabolic activity of clones. Likewise for the levels of SOD at a given water content. A water content of 30% showed relatively the same SOD activity as 60% and 90% in all observations. This indicates the SOD formed in low levels in the observations and therefore cannot be used as a marker of tolerance for these tested clones. SOD is one of the key components of cell protection against oxidative stress. The SOD has three different isoenzymes distributed between organelles. Cu/Zn-SOD is predominantly located in the chloroplasts, cytosol, and peroxisomes, whereas FeSOD and MnSOD are mostly found in chloroplasts and mitochondria, respectively. ^47^ POD and SOD activities increased sharply in rubber seedlings after being subject to drought stress. This suggests that the photosynthetic activity and lipid integrity of the cell membranes are rapidly attenuated by drought stress. SODs are metalloenzymes that play an important role in ROS reactions, or, in other words, are able to neutralize the negative effects of ROS. The decrease in substrate binding affinity to SOD as well as a decrease in one isozyme band of SOD under drought conditions may be responsible for the resistance. Plants that have a higher induced SOD activity show more tolerance to abiotic stresses. Numerous studies have shown that plants are able to better eliminate the negative effects of ROS produced under stressful situations when their SOD activity is higher, provided there are more SOD isoenzymes present.

POD had low values in all six observations of some clones. The low POD indicated that the effect of some water content percentages given during the six observations on several different clones did not have a significant effect. This indicates that the POD characteristics cannot be used as a reference of plant tolerance to drought stress. Plants that produce more POD under conditions of drought stress will be able to survive by eliminating the effects of ROS. In general, the activity of POD and other antioxidant enzymes will automatically have a higher value in tolerant clones/varieties and will have a lower value in susceptible clones/varieties. This indicates that drought tolerant clones/varieties will be more efficient in removing H _2_O _2_ to produce optimal protection. Tolerance of some genotypes to environmental stresses has been associated with higher antioxidant enzyme activity. Drought-tolerant species of pigeon pea ( *Cajanus cajan*), ^48^ wheat ( *Triticum aestivum*), ^49^^,^^50^ and black bean ( *Phaseolus mungo*) ^47^ have higher SOD, POD, and CAT activities than drought-sensitive species. The results of this study indicate that ROS enzymes, which play a crucial role in the drought-tolerance mechanism under the drought treatment, have been identified in clones IRR 425, IRR 428, IRR 429, IRR 434, IRR 440, RRIC 100, and BPM 24 as scions. Based on the findings, several analyses have been carried out on physiological characteristics to determine the effects of water content on a greenhouse scale.

## Conclusions

The tolerance ability of the IRR 400 series rubber clones to drought stress was determined by observing the two characteristics of total sugar and proline levels. Furthermore, chlorophyll a, b, and total, H _2_O _2_, APX SOD, and POD should not be used as markers of drought stress tolerance in rubber trees. The concentrations of total sugar and proline were higher when the plants were treated with a lower water content.

## Data Availability

This project contains the following underlying data: Figshare: Data set: Physiological Characters of IRR 400 Series Rubber Clones ( *Hevea brasiliensis* Muell. Arg.) on Drought Stress. https://doi.org/10.6084/m9.figshare.21708230.v2 ^51^
- Biochemistry characters.xlsx (datasets for total sugar, chlorophyll (a, b, total), proline, H _2_O _2_, APX, SOD, POD) Figshare: Data set: Physiological Characters of IRR 400 Series Rubber Clones ( *Hevea brasiliensis* Muell. Arg.) on Drought Stress. https://doi.org/10.6084/m9.figshare.21708275.v2 ^52^
- Polyortogonal_test.xlsx (datasets for total sugar, chlorophyll (a, b, total), proline, H _2_O _2_, APX, SOD, POD) Figshare: Data set: Physiological Characters of IRR 400 Series Rubber Clones ( *Hevea brasiliensis* Muell. Arg.) on Drought Stress. https://doi.org/10.6084/m9.figshare.21645116.v5 ^53^ This project contains the following extended data:
- Suplementary materials.docx (supplementary Tables 1–9):
•
Table 1. The sugar total of IRR 400 series, RRIC 100, BPM 24 and some water content (30%, 60%, 90% FC) of six 3 observations. •
Table 2. The proline of IRR 400 series, RRIC 100, BPM 24 and some water content (30%, 60%, 90% FC) on six observations. •
Table 3. The chlorophyll-a of IRR 400 series, RRIC 100, BPM 24 and some water content (30%, 60%, 90% FC) on six 14 observations. •
Table 4. The chlorophyll-b IRR 400 series, RRIC 100, BPM 24 and some water content (30%, 60%, 90% FC) on six 20 observations. •
Table 5. The total chlorophyll of IRR 400 series, RRIC 100. BPM 24 and some water content (30%, 60%, 90% FC) on six 26 observations. •
Table 6. The hydrogen peroxidase (H _2_O _2_) of IRR 400 series, RRIC 100, BPM 24 and some water content (30%, 60%, 90% 32 FC) on six observations. •
Table 7. The ascorbate peroxidase/APX of IRR 400 series, RRIC 100, BPM 24 and some water content (30%, 60%, 90% 38 FC) on six observations. •
Table 8. The superoxide dismutase/SOD of IRR 400 series, RRIC 100, BPM 24 and some water content (30%, 60%, 90% 44 FC) on six observations. •
Table 9. The peroxide dismutase/POD of IRR 400 series, RRIC 100, BPM 24 and some water content (30%, 60%, 90% 50 FC) on six observations. Data are available under the terms of the Creative Commons Attribution 4.0 International license (CC-BY 4.0).
