# Physiological characteristics of IRR 400 series rubber clones ( *Hevea brasiliensis* Muell. Arg.) under drought stress

**DOI:** 10.12688/f1000research.129421.4

**Published:** 2023-12-04

**Authors:** Syarifah Aini Pasaribu, Mohammad Basyuni, Edison Purba, Yaya Hasanah

**Affiliations:** 1 Unit Research Sungei Putih, Indonesian Rubber Research Institute, Galang, Deliserdang, North Sumatra, 20585, Indonesia; 2 Center of Excellence for Mangrove, Universitas Sumatera Utara, Medan, 20155, Indonesia; 3 Department of Forestry, Universitas Sumatera Utara, Medan, North Sumatra, 20155, Indonesia; 4 Department of Agrotechnology, Universitas Sumatera Utara, Medan, North Sumatra, 20155, Indonesia

**Keywords:** rubber, drought stress, water content, adaptation, abiotic stress

## Abstract

**Background:**

Drought stress is one of the main causes of plant death. Strategies for plants survival are morphological adaptations, specific signaling pathways, and tolerance mechanisms. Rubber plantations have many uses, such as foreign exchange sources, job sources, forest revitalization, and a source of alternative wood for building materials and furniture. The rubber plant’s response to drought stress is a complex biological process. A tolerant rubber clone in a dry area is the right approach. The present study aimed to identify drought tolerant traits in order to select or identify drought-tolerant clones at juvenile stage.

**Methods:**

The first factor examined for this work was clones (IRR 425, IRR 428, IRR 429, IRR 434, IRR 440, RRIC 100, and BPM 24) and the second factor was water content (30%, 60%, and 90%). The study was arranged on a factorial randomized block design and repeated three times. Characteristics observed were total sugar (µM), proline (mg/L), chlorophyll a, b, total (µg/mL), hydrogen peroxidase (µmol/g), ascorbate peroxidase (unit/mg), superoxide dismutase (unit/mg), and peroxide dismutase (unit/mg).

**Results:**

The tolerance ability of the IRR 400 series rubber clones to drought stress was determined by observing the characteristics of sugar total and proline. The concentration of total sugar and proline were higher when the plant was treated with a lower water content. The selected clones tolerant to drought stress are RR 425 and IR 434 with high total sugar content and proline. Other characteristics, namely chlorophyll a, b, and total, as well as hydrogen peroxidase, ascorbate peroxidase, super oxide dismutase, peroxide dismutase, cannot be used as selection characteristics for this study.

**Conclusions:**

This drought study of IRR 400 clones with varying water content percentages illustrated that the total sugar and proline characteristics could be used to distinguish tolerance levels from other observed characteristics.

## Introduction

In rubber plants, drought can cause a delayed maturation phase, short tapping period, slow latex flow, dry latex, increased dry tapping grooves, and even tree death. ^1^ Drought is one of the main abiotic stresses affecting yield and productivity in almost all crops. ^2^ Hence, its significance overshadows that of other environmental factors because it interferes with plant growth and development and disrupts production and performance. Water is an important component of the protoplasm and makes up 85%–90% of the total weight of the plant tissue. Water is also a vital reagent in photosynthesis and hydrolysis reactions. Additionally, it acts as a solvent for salts, gases, and other substances transported between cell tissues to maintain cell growth and leaf shape stability. ^3^

One of the primary sources of natural rubber producing plants was found in the Amazon basin, South America. ^4^ Optimal conditions for the growth of rubber plants include high temperature (28 ± 2 °C), high humidity, and rainfall of 2000–4000 mm/year. ^5^ Rubber plantations in marginal areas, such as Central India, southern China, northern and northeastern Thailand, and eastern Indonesia, experience occasional drought. Indonesia has a wide drought area of about 122.1 million ha, which is not optimally exploited due to limited water resources.

The response caused by drought with regard to plant is quite complex because it involves changes in morphology, physiology, and metabolism. The initial response to drought stress is the loss of turgor pressure, which results in reduced growth rate and leaf senescence. Drought changes the source–sink relationship and affects the translocation of photosynthate to produce fruit quickly for certain crops. ^6^ The fastest response to a water deficit is the stomatal closure to protect plants from water shortages. Water deficit results in abscisic acid (ABA) biosynthesis, which triggers stomatal closure and causes a decrease in intracellular CO _2_ levels and the inhibition of photosynthesis. ^7^ Water shortages do not always promote these responses in all plant species. Lack of intracellular CO _2_ due to prolonged stomatal closure leads to the accumulation of reactive oxygen and nitrogen species, which damages the photosynthetic apparatus. ^8^ Besides that, the presence of osmoprotectants, such as proline, trehalose sugar, glycine betaine, donomitol, and mannitol maintain the growth and productivity of a plant experiencing drought stress. ^9^ ^–^ ^11^ The presence of antioxidant enzymes, such as superoxide dismutase (SOD), catalase (CAT), ascorbate peroxidase (APX), and glutathione reductase (GR), in cellular and cytoplasmic organelles play an important role in the detoxification of these reactive oxygen species (ROS) and enable plant cells to activate various stress sensors, which will then activate various signal paths.

Inhibited growth is a typical symptom of drought stress. ^12^ The consequent physiological, biochemical, and molecular changes affect various cellular processes, thereby reducing the quantity and quality of the plant yield. The combined rise in atmospheric CO _2_ concentrations and global air tempeature may significantly alter plant water use. ^13^ Pasaribu et al, reported based on bio-informatics, there are 20 proteins related to drought stress in rubber plants. ^14^ This study aims to identify drought tolerant traits in order to select or identify drought-tolerant clones at juvenile stage.

## Methods

### Study area

Analysis of physiological characteristics was carried out at the Physiology and Protection Laboratory of the Unit Research SungeiPutih, Galang, Deli Serdang, North Sumatra. The study was carried out in a greenhouse during June 2020–May 2021. The materials used were red-yellow podzolic soil (water content = 3.9, pH = 4.5, C-organic = 0.92, N = 0.15, P _2_O _5_ = 2.13), compound fertilizer, Dithane M-45, and Triko-SP Plus. The tools used included polybags (18 × 35 cm and 50×60 cm), a hoe, a soil sieve, bucket, watering can, a 100 kg UK scale, an analytical balance, object glass, deck glass, binocular microscope, water bath, vortex, UV spectrophotometer, filter paper, test tube, gloves, mask, tissue, distilled water, mortar, beaker, micropipettes 1 ml and 100 μl, stirrer, 15-watt lamp, microcentrifuge, microwave, and others.

### Stages of growth and development of the clones

The plants age was 6-12 months, planted in polybags measuring 50 × 60 cm filled with 30 kg ultisol soil. The samples used were normal leaves and not affected by leaf fall disease such as Oidium, Colletotrichum, Corynespora and Pestalotiopsis. The leaves were taken between at 08.00-09.00 AM. The average temperature in the greenhouse during the six months observation was 25.9 °C (morning), 33.9 °C (midday) and 30.4 °C (afternoon). The average temperature outside the greenhouse was 22.9 C (morning), 28.1 °C (midday) and 25.5 °C (afternoon). The average humidity was 92.6% (morning), 62.9% (midday) and 69.2% (afternoon).

### Experimental design

The study was arranged based on a factorial randomized block design (RBD). The first factor was the type of clone, consisting of seven types, namely C1: IRR 425, C2: IRR 428, C3: IRR 429, C4: IRR 434, C5: IRR 440, C6: RRIC 100, and C7: BPM 24. The second factor was water content, consisting of three levels, namely: W1: 30%, W2: 60%, and W3: 90%. Each experimental unit was repeated three times, and as many as 63 samples were observed.

### Data analysis

Observations were carried out six times on physiological characteristics, with an intervals of three weeks. If the test of variance obtained significantly different treatments, then the Tukey distance test of 0.5% was carried out. ^15^ The characteristics observed were total sugar content, ^16^ chlorophyll a, b, and total, ^17^ proline, ^18^ ^–^ ^21^ super peroxidase dismutase (SOD), ^22^ peroxidase dismutase (POD), ^23^ APX enzyme, ^24^ and hydrogen peroxide (H _2_O _2_). ^22^

A step-by-step description of the procedure to analyze sugar content, proline, chlorophyll a, b, and total, SOD, POD, H _2_O _2_, and APX has been deposited in protocols.io and is available at dx.doi.org/10.17504/protocols.io.5jyl8je1dg2w/v1.

## Results

### Total sugar content (μM)

The total sugar content analysis showed a significant effect in all the observations except the first one ( Table 1).

**Table 1.** The total sugar content (μM) in different clones, different water content (%), and interactions between clone type and water content (%).

Treatments	Total sugar content (μM)											
	Period to											
	1		2		3		4		5		6	
**Clones (C)**												
IRR 425	158.99	a	65.53	d	90.74	b	105.17	b	198.38	b	175.77	b
IRR 428	189.62	a	97.27	b	92.06	b	127.02	ab	190.27	bc	136.85	e
IRR 429	190.39	a	122.26	a	98.64	b	146.21	a	221.09	a	140.27	e
IRR 434	178.26	a	96.40	b	92.99	b	132.04	ab	190.12	bc	160.50	c
IRR 440	156.32	a	77.94	cd	96.55	b	127.07	ab	183.62	c	151.19	d
RRIC 100	198.05	a	129.34	a	11.90	a	129.26	ab	193.01	bc	184.73	a
BPM 24	178.27	a	87.36	bc	73.28	c	158.75	a	192.28	bc	166.85	c
**Water content (WC)**												
30%	188.24	a	111.68	a	116.79	a	151.64	a	200.57	a	178.83	a
60%	174.91	a	93.05	b	88.02	b	132.12	b	180.29	b	156.78	b
90%	172.51	a	85.03	c	77.69	c	112.89	c	205.76	c	142.74	c
**Interactions C × WC**												
C1WC1	172.96	a	73.56	ghij	94.76	cdefg	117.88	bc	219.11	a	197.64	abc
C1WC2	166.06	a	63.38	ghij	106.65	bcdef	94.52	c	169.08	bcd	186.03	bc
C1WC3	137.94	a	54.66	j	70.81	g	103.12	c	206.96	ab	143.38	fg
C2WC1	186.45	a	107.60	cdef	111.73	bcde	161.67	abc	181.09	bcd	121.82	h
C2WC2	145.41	a	123.18	bc	92.72	cdefg	113.25	bc	180.51	bcd	143.02	fg
C2WC3	236.99	a	61.03	ij	71.72	g	106.15	bc	209.22	ab	145.70	fg
C3WC1	232.61	a	149.85	a	125.52	ab	206.06	a	227.64	a	164.65	de
C3WC2	168.13	a	111.00	cde	84.10	defg	127.88	abc	213.41	ab	134.68	fgh
C3WC3	170.43	a	105.93	cdef	86.32	efg	104.68	bc	222.21	a	121.48	h
C4WC1	178.63	a	107.71	cdef	122.59	abc	147.23	abc	157.15	d	183.03	cd
C4WC2	178.71	a	87.76	efgh	73.33	g	141.14	abc	208.45	ab	165.46	de
C4WC3	177.42	a	93.72	defg	83.05	efg	107.73	bc	204.76	ab	133.02	gh
C5WC1	164.30	a	83.03	ghij	135.69	ab	147.12	abc	209.03	ab	164.55	e
C5WC2	166.67	a	74.74	fghi	76.94	fg	128.34	abc	171.20	cd	152.69	ef
C5WC3	137.98	a	76.06	fghij	77.01	fg	105.74	bc	170.62	cd	136.32	fgh
C6WC1	210.53	a	148.33	ab	151.18	a	146.51	abc	215.12	a	215.48	a
C6WC2	216.43	a	119.44	cd	116.71	bcd	135.66	abc	152.74	d	186.18	bc
C6WC3	167.18	a	120.25	c	76.82	fg	105.62	bc	211.17	ab	152.52	ef
C7WC1	172.19	a	111.64	cde	76.05	fg	135.00	abc	194.85	abc	204.64	ab
C7WC2	182.99	a	66.88	hij	65.70	g	184.08	ab	166.64	cd	129.11	gh
C7WC3	179.63	a	83.56	fghi	78.09	g	157.17	abc	215.35	a	166.80	de

Notes: C1: IRR 425, C2: IRR 428, C3: IRR 429, C4: IRR 434, C5: IRR 440, C6: RRIC 100, C7: BPM 24, WC1: 30%, WC2: 60%, WC3: 90%. Data is represented by ±SD value. The same letters were not significantly different from each other (P<0.05) using Tukey's test.

The total sugar content in the six observations carried out on tested clones was consistent. The RRIC 100 clone had the highest total sugar content four times, and the IRR 425 clone had the lowest four times.

The total sugar content analysis at different water levels generally showed a significant effect, except for the initial observation. This indicates that water content affects the total sugar content of the tested clones ( Table 1).

Analysis of the total sugar content due to the interaction between the clone type and water content level (30%, 60%, and 90%) showed significant differences, except in the first observation ( Table 1).

What is interesting about these results is that the highest accumulation of total sugar was seen in the application of 30% water content. Meanwhile, the effects were quite diverse across the different clone types. The IRR 429 had the highest total sugar in three observations (second, fourth, and fifth). The RRIC 100 had the highest total sugar in two observations (third and sixth). The two clones, RRIC 100 and IRR 429, also had the lowest total sugar in the fifth and sixth observations, respectively.

**Figure 1.**
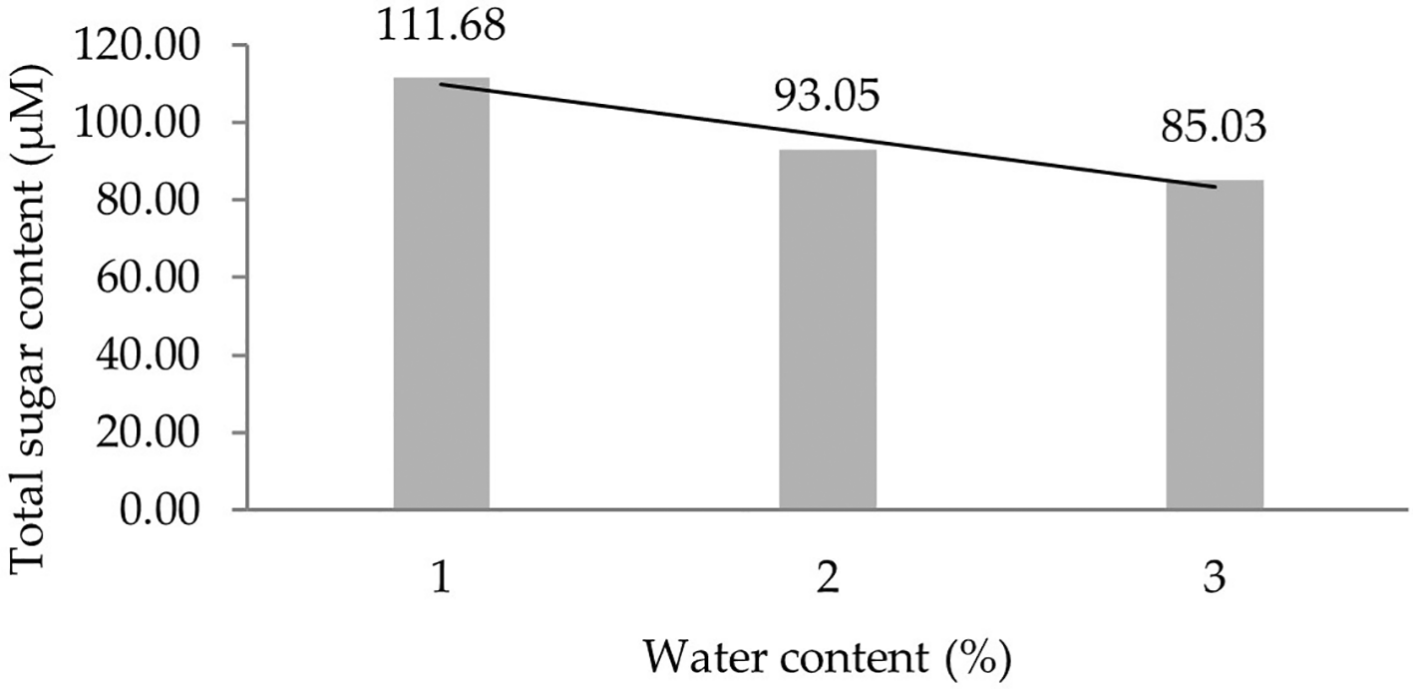
Pattern of total sugar content linear curve as a result of orthogonal polynomial analysis. 1: 30%; 2: 60%; 3: 90%.

Two forms of polynomial curves can be the effect of water content and can be shown by the orthogonal polynomial regression obtained from three levels of water content, namely linear and cubic curves. The results of the analysis show that the linear curve shows a real effect. Figure 1 presents the linear curve regression pattern formed in detail. It demonstrates that the lower the water content added to the growing media, the higher the total sugar content derived from the leaf analysis of several rubber clones of IRR 400 series, RRIC 100, and BPM 24.

### Proline (mg g ^-1^)

Table 2 depicts the proline analysis of clone types treated with different water content levels and shows significantly different effects in all observations.

**Table 2.** Proline levels (mg g ^-1^) in different clones, different water content (%), and interactions between clone type and water content (%).

Treatments	Proline (mg g ^-1^)											
	Period to											
	1		2		3		4		5		6	
**Clones (C)**												
IRR 425	11.16	a	9.25	a	11.47	cd	8.11	a	8.95	a	6.86	a
IRR 428	10.38	a	5.74	c	14.93	a	7.61	ab	7.83	b	6.64	ab
IRR 429	10.51	a	9.27	a	11.78	cd	7.14	abc	7.91	b	6.56	ab
IRR 434	7.38	b	7.14	bc	11.92	bc	7.89	a	4.44	d	6.74	a
IRR 440	6.23	b	7.67	ab	8.76	e	6.25	c	5.33	cd	5.69	b
RRIC 100	5.89	b	6.13	bc	9.99	de	6.29	c	5.57	c	5.99	ab
BPM 24	7.02	b	6.96	bc	13.66	ab	6.80	bc	7.30	b	5.98	ab
**Water content (WC)**												
30%	11.02	a	11.27	a	15.17	a	8.30	b	8.95	a	7.97	a
60%	8.88	b	6.92	b	10.78	b	6.99	a	6.77	b	6.36	b
90%	5.21	c	4.20	c	9.32	c	6.18	b	4.56	b	4.72	a
**Interactions C × WC**												
C1WC1	13.82	a	13.65	a	17.04	ab	9.43	abcd	12.32	a	8.29	g
C1WC2	13.57	a	8.21	efgh	8.47	ghi	8.00	a	9.10	efg	7.98	abc
C1WC3	6.09	cde	5.91	defg	8.19	ghi	6.90	bcdefg	5.43	cd	4.30	ab
C2WC1	13.26	a	7.54	defgh	15.69	abc	9.19	bcdefg	10.26	bc	9.13	fg
C2WC2	11.25	ab	4.89	ghi	15.51	abc	6.87	a	6.92	e	6.23	a
C2WC3	6.64	cde	4.79	ghij	13.60	bcd	6.78	bcdefg	6.31	efg	4.55	cdefg
C3WC1	13.55	a	14.87	a	13.53	cdefg	7.76	abcde	11.77	fg	8.69	a
C3WC2	13.28	a	7.63	fghi	12.12	bcde	7.42	defg	7.19	ab	6.10	fg
C3WC3	4.70	def	5.31	defgh	9.70	efgh	6.25	abcdef	4.78	de	4.62	cdefg
C4WC1	8.40	bcd	12.38	hij	17.61	a	8.75	cdefg	5.47	efg	8.42	abcde
C4WC2	6.94	cde	4.85	ab	9.36	fgh	8.41	abc	5.28	hi	7.11	fg
C4WC3	6.79	cde	4.45	hij	8.79	ghi	6.50	ab	2.58	efg	4.69	ab
C5WC1	7.17	cde	11.74	bcde	11.66	defg	7.48	efg	6.88	e	7.14	efg
C5WC2	6.60	cde	8.93	abc	9.27	fgh	5.72	abcdef	6.68	ef	5.22	abcde
C5WC3	4.91	edef	2.33	ij	5.35	i	5.55	fg	2.42	i	4.69	fg
C6WC1	12.10	ab	10.11	defgh	15.94	abc	8.11	g	6.73	ef	6.39	bcdef
C6WC2	4.15	f	6.95	bcd	7.46	hi	5.61	abcd	5.52	efg	5.81	defg
C6WC3	1.42	ef	1.32	j	6.57	hi	5.14	fg	4.46	gh	5.77	defg
C7WC1	8.84	bc	8.62	cdef	14.71	abcd	7.39	abcdef	9.24	c	7.46	abcd
C7WC2	6.35	cde	6.89	fghi	13.25	bcde	6.89	bcdefg	6.71	ef	6.07	cdefg
C7WC3	5.89	cde	5.29	defgh	13.10	cdef	6.11	defg	5.97	efg	4.41	fg

Notes: C1: IRR 425, C2: IRR 428, C3: IRR 429, C4: IRR 434, C5: IRR 440, C6: RRIC 100, C7: BPM 24, WC1: 30%, WC2: 60%, WC3: 90%. Data is represented by ±SD value. The same letters were not significantly different from each other (P<0.05) using Tukey's test.

The results of proline analysis at different water content levels showed significantly different effects in all observations (see Table 2).

The proline analysis caused by the interaction between rubber clones IRR 400 series, RRIC 100, and BPM 24 and given water content (30%, 60%, 90%) showed significantly different effects in all observations, as displayed in Table 2.

**Figure 2.**
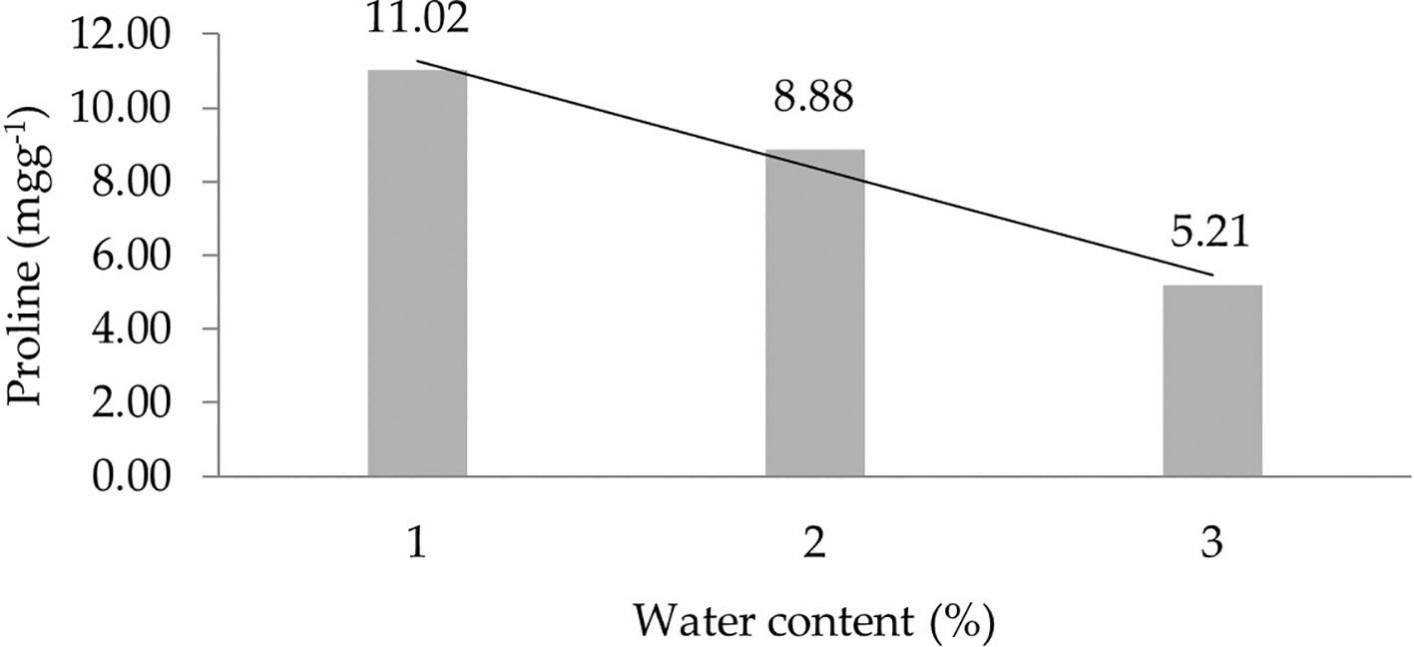
Pattern of proline linear curve as a result of orthogonal polynomial analysis. 1: 30%; 2: 60%; 3: 90%.

The assessment of orthogonal polynomial regression showed a linear curve, where the water content at the 30% level had the highest proline value. The orthogonal polynomial linear curve pattern of proline characteristics of several rubbers, namely, IRR 400 series, RRIC 100, and BPM 24, is shown in Figure 2.

### Chlorophyll a (μg mg ^-1^)

The chlorophyll-a analysis on the different clone types showed a significant effect, except for the first observation, as displayed in Table 3.

**Table 3.** Chlorophyll a levels (μg mg ^-1^) in different clones, different water content (%), and interactions between clones and water content (%).

Treatments	Chlorophyll-a (μg mg ^-1^)											
	Period to											
	1		2		3		4		5		6	
**Clones (C)**												
IRR 425	0.25	a	0.60	a	0.46	C	0.60	b	0.62	a	0.72	a
IRR 428	0.23	a	0.48	b	0.42	d	0.48	d	0.50	b	0.48	bc
IRR 429	0.24	a	0.59	a	0.51	b	0.55	c	0.32	d	0.37	d
IRR 434	0.24	a	0.47	b	0.34	f	0.47	d	0.48	bc	0.38	d
IRR 440	0.23	a	0.39	c	0.38	e	0.37	e	0.28	d	0.48	bc
RRIC 100	0.25	a	0.61	a	0.55	a	0.73	a	0.49	b	0.44	c
BPM 24	0.22	a	0.46	b	0.41	de	0.49	d	0.45	c	0.52	b
**Water content (WC)**												
30%	0.28	a	0.64	a	0.54	a	0.67	a	0.57	a	0.59	a
60%	0.23	b	0.51	b	0.44	b	0.48	b	0.42	b	0.47	b
90%	0.20	c	0.40	c	0.33	c	0.43	c	0.36	c	0.39	c
**Interactions C × WC**												
C1WC1	0.32	a	0.74	a	0.66	a	0.74	bc	0.76	a	0.85	a
C1WC2	0.24	ab	0.64	c	0.47	efg	0.64	de	0.56	cde	0.67	b
C1WC3	0.18	b	0.42	efg	0.26	l	0.42	g	0.53	efg	0.63	bc
C2WC1	0.26	a	0.67	bc	0.48	def	0.67	cd	0.65	bc	0.55	cd
C2WC2	0.24	ab	0.42	efg	0.41	fgh	0.42	g	0.44	ghi	0.46	ef
C2WC3	0.19	ab	0.35	gh	0.35	hijk	0.34	h	0.41	hij	0.43	fg
C3WC1	0.29	a	0.75	a	0.63	ab	0.75	b	0.45	ghi	0.47	ef
C3WC2	0.23	ab	0.56	d	0.57	bc	0.46	g	0.28	kl	0.34	h
C3WC3	0.19	ab	0.46	e	0.34	ijk	0.44	g	0.23	l	0.30	h
C4WC1	0.27	ab	0.56	d	0.36	hij	0.56	f	0.62	bcd	0.46	ef
C4WC2	0.22	ab	0.44	ef	0.33	ijkl	0.44	g	0.45	ghi	0.36	gh
C4WC3	0.22	ab	0.43	ef	0.32	jkl	0.43	g	0.37	ij	0.33	h
C5WC1	0.26	ab	0.45	e	0.46	efg	0.45	g	0.34	jk	0.57	cd
C5WC2	0.23	ab	0.37	fgh	0.40	ghi	0.34	h	0.26	kl	0.53	de
C5WC3	0.22	ab	0.35	h	0.28	kl	0.31	h	0.24	l	0.33	h
C6WC1	0.28	ab	0.73	ab	0.65	ab	0.93	a	0.65	b	0.55	cd
C6WC2	0.24	ab	0.64	c	0.54	cd	0.63	def	0.46	fgh	0.43	fg
C6WC3	0.22	ab	0.45	e	0.47	defg	0.62	def	0.37	ij	0.35	h
C7WC1	0.25	ab	0.55	d	0.53	cde	0.57	ef	0.55	def	0.67	b
C7WC2	0.21	ab	0.47	e	0.38	hij	0.45	g	0.46	fgh	0.52	de
C7WC3	0.19	ab	0.36	h	0.33	ijkl	0.44	g	0.34	jk	0.36	gh

Notes: C1: IRR 425, C2: IRR 428, C3: IRR 429, C4: IRR 434, C5: IRR 440, C6: RRIC 100, C7: BPM 24, WC1: 30%, WC2: 60%, WC3: 90%. Data is represented by ±SD value. The same letters were not significantly different from each other (P<0.05) using Tukey's test.

Table 3 shows the chlorophyll-a analysis at different water contents, which demonstrated a significant effect in all six observations.

Analysis of chlorophyll-a levels due to the interaction between clones and water content (30%, 60%, 90%) showed significant differences in all six observations ( Table 3).

### Chlorophyll b (μg mg ^-1^)

The results of the chlorophyll b analysis with different clone types showed significantly different results, except for the first observation ( Table 4).

**Table 4.** Chlorophyll b levels (μg mg ^-1^) in different clones, different water content (%) and interactions between clones and water content (%).

Treatments	Chlorophyll-b (μg mg ^-1^)											
	Period to											
	1		2		3		4		5		6	
**Clones (C)**												
IRR 425	0.24	a	0.57	a	0.44	b	0.57	a	0.55	ab	0.52	ab
IRR 428	0.22	a	0.50	b	0.42	b	0.51	b	0.52	b	0.42	c
IRR 429	0.21	a	0.53	ab	0.33	c	0.55	ab	0.46	c	0.37	d
IRR 434	0.23	a	0.30	e	0.33	c	0.34	d	0.47	c	0.39	cd
IRR 440	0.23	a	0.45	c	0.44	cb	0.41	c	0.46	c	0.56	a
RRIC 100	0.23	a	0.42	cd	0.45	b	0.55	ab	0.48	c	0.49	b
BPM 24	0.20	a	0.40	d	0.54	a	0.55	ab	0.56	a	0.55	a
**Water content (WC)**												
30%	0.25	a	0.52	a	0.49	a	0.60	a	0.61	a	0.59	a
60%	0.22	b	0.45	b	0.42	b	0.48	b	0.49	b	0.44	b
90%	0.20	c	0.38	c	0.35	c	0.41	c	0.39	c	0.38	c
**Interactions C × WC**												
C1WC1	0.26	abc	0.70	a	0.57	ab	0.70	ab	0.66	ab	0.74	a
C1WC2	0.26	abc	0.55	cd	0.43	cd	0.56	cde	0.63	b	0.46	bcd
C1WC3	0.20	ab	0.46	defg	0.33	e	0.46	efghi	0.36	gh	0.36	efgh
C2WC1	0.25	abc	0.64	ab	0.45	c	0.64	abc	0.64	b	0.54	b
C2WC2	0.23	abc	0.47	defg	0.42	cd	0.47	efgh	0.55	c	0.37	defgh
C2WC3	0.19	c	0.39	ghijk	0.38	cde	0.42	ghij	0.37	gh	0.35	fgh
C3WC1	0.28	a	0.57	bc	0.37	cde	0.61	bcd	0.54	cde	0.46	bcde
C3WC2	0.18	bc	0.53	cde	0.32	e	0.55	cde	0.47	cdef	0.34	gh
C3WC3	0.17	bc	0.48	defg	0.31	e	0.48	efgh	0.36	gh	0.30	h
C4WC1	0.26	abc	0.33	kl	0.34	e	0.37	ijk	0.54	cd	0.45	bcdef
C4WC2	0.21	abc	0.30	kl	0.33	e	0.34	jk	0.46	ef	0.37	defgh
C4WC3	0.21	abc	0.26	l	0.30	e	0.30	k	0.42	fgh	0.35	gh
C5WC1	0.26	ab	0.52	cdef	0.54	b	0.52	defg	0.63	b	0.69	a
C5WC2	0.22	abc	0.46	defg	0.44	c	0.37	ijk	0.41	fgh	0.54	b
C5WC3	0.22	abc	0.36	ijk	0.34	e	0.35	jk	0.35	h	0.45	bcde
C6WC1	0.26	abc	0.45	efgh	0.55	b	0.73	a	0.55	c	0.54	b
C6WC2	0.22	abc	0.43	fghij	0.44	c	0.55	cde	0.46	def	0.48	bc
C6WC3	0.21	abc	0.36	hijk	0.36	de	0.38	hijk	0.43	Fg	0.43	cdefg
C7WC1	0.21	abc	0.45	efghi	0.64	a	0.66	ab	0.73	a	0.70	a
C7WC2	0.20	abc	0.40	ghijk	0.53	b	0.53	def	0.48	cdef	0.54	b
C7WC3	0.19	abc	0.35	jkl	0.44	c	0.45	fghi	0.47	cdef	0.41	cdefg

Notes: C1: IRR 425, C2: IRR 428, C3: IRR 429, C4: IRR 434, C5: IRR 440, C6: RRIC 100, C7: BPM 24, WC1: 30%, WC2: 60%, WC3: 90%. Data is represented by ±SD value. The same letters were not significantly different from each other (P<0.05) using Tukey's test.

The results of the analysis of chlorophyll b levels at the given water contents showed a significant effect in all six observations ( Table 4).

The analysis of chlorophyll b levels due to the interaction between clones and water content (30%, 60%, 90%) showed significant differences in all six observations, as shown in Table 4.

**Figure 3.**
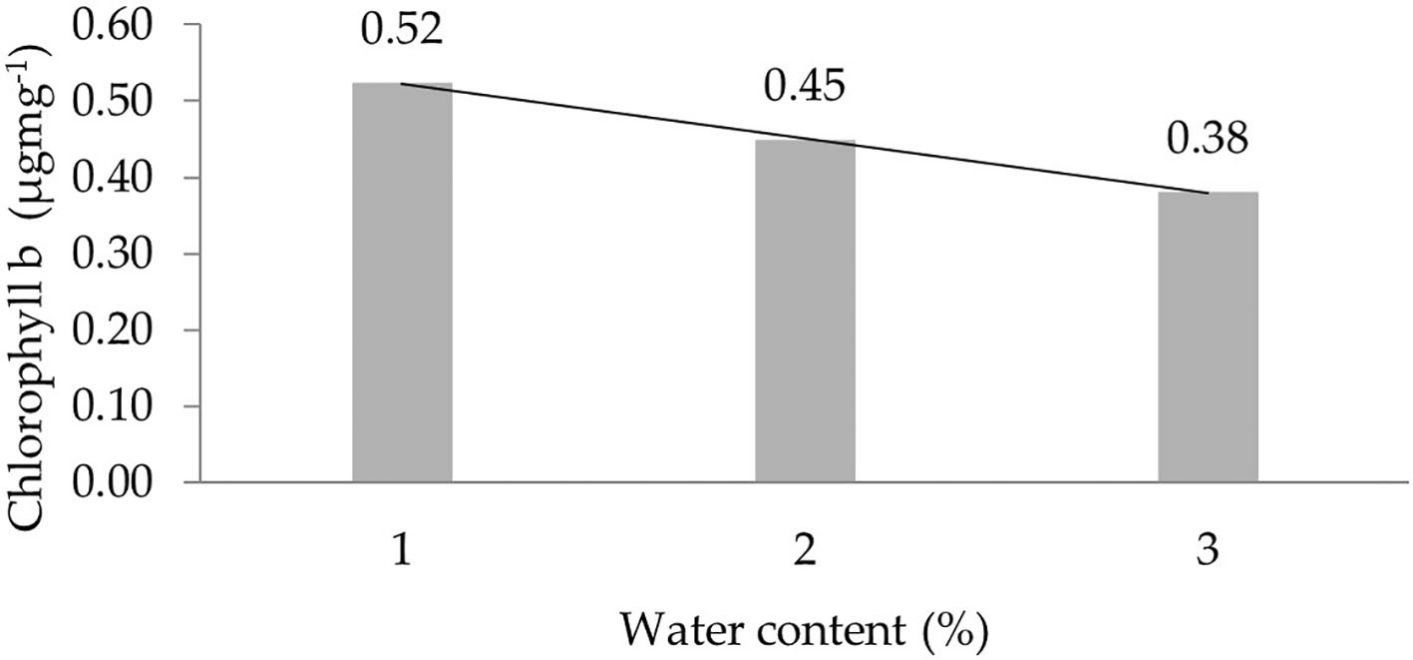
Pattern of chlorophyll b linear curve as a result of orthogonal polynomial analysis. 1: 30%; 2: 60%; 3: 90%.

The assessment of the orthogonal polynomial regression showed a linear curve, where the water content at the 30% level had the highest chlorophyll b value. The orthogonal polynomial linear curve pattern of the chlorophyll b characteristics of several rubber clones of IRR 400 series, RRIC 100, and BPM 24 can be seen in Figure 3.

### Total chlorophyll content (μg mg ^-1^)

The analysis results of total chlorophyll content with different clone types showed significantly different results except for one observation ( Table 5).

**Table 5.** Total chlorophyll content levels (μg mg ^-1^) in different clones, different water content (%), and interactions between clones and water content (%).

Treatments	Total chlorophyll content (μg mg ^-1^)											
	Period to											
	1		2		3		4		5		6	
**Clones (C)**												
IRR 425	0.48	a	1.17	a	0.90	b	1.17	b	1.17	a	1.24	a
IRR 428	0.46	a	0.98	b	0.83	c	0.99	d	1.02	b	0.90	c
IRR 429	0.45	a	1.12	a	0.84	c	1.10	c	0.78	d	0.74	d
IRR 434	0.46	a	0.77	d	0.66	d	0.81	e	0.95	c	0.77	d
IRR 440	0.47	a	0.84	c	0.82	c	0.78	e	0.74	e	1.04	b
RRIC 100	0.47	a	1.02	b	1.00	a	1.28	a	0.97	bc	0.93	c
BPM 24	0.42	a	0.86	c	0.95	b	1.03	d	1.01	b	1.06	b
**Water content (WC)**												
30%	0.53	a	1.16	a	1.03	a	1.27	a	1.19	a	0.90	a
60%	0.45	b	0.96	b	0.86	b	0.96	b	0.91	b	0.74	b
90%	0.40	c	0.78	c	0.69	c	0.83	c	0.75	c	0.77	c
**Interactions C × WC**												
C1WC1	0.57	a	1.44	a	1.23	a	1.44	b	1.42	a	1.59	a
C1WC2	0.50	abcd	1.19	cd	0.90	bcd	1.19	c	1.19	bc	1.13	cd
C1WC3	0.38	d	0.88	hij	0.59	h	0.88	efg	0.89	efg	0.99	ef
C2WC1	0.51	abcd	1.31	bc	0.93	bcd	1.31	bc	1.29	b	1.09	de
C2WC2	0.48	abcd	0.89	hij	0.84	de	0.89	defg	0.99	d	0.83	gh
C2WC3	0.38	bcd	0.74	klm	0.74	efg	0.76	gh	0.78	h	0.78	ghi
C3WC1	0.57	a	1.33	ab	0.99	bc	1.37	b	0.98	de	0.92	fg
C3WC2	0.41	abcd	1.09	def	0.89	cd	1.01	d	0.75	def	0.69	hij
C3WC3	0.36	cd	0.94	hij	0.64	gh	0.91	def	0.60	gh	0.60	j
C4WC1	0.53	abcd	0.89	hij	0.70	fgh	0.92	de	1.16	c	0.90	fg
C4WC2	0.43	abcd	0.73	klm	0.66	fgh	0.78	fgh	0.91	def	0.73	hij
C4WC3	0.43	abcd	0.70	m	0.62	gh	0.74	h	0.79	gh	0.68	ij
C5WC1	0.52	abc	0.97	ghi	1.00	b	0.97	de	0.97	de	1.26	bc
C5WC2	0.45	abcd	0.83	jk	0.83	de	0.71	h	0.68	i	1.07	de
C5WC3	0.43	abcd	0.71	lm	0.62	gh	0.66	h	0.58	i	0.79	ghi
C6WC1	0.54	ab	1.19	de	1.20	a	1.67	a	1.20	bc	1.09	de
C6WC2	0.45	abcd	1.07	efg	0.98	bc	1.18	c	0.93	de	0.91	fg
C6WC3	0.43	abcd	0.81	jkl	0.82	de	1.01	de	0.79	gh	0.78	ghi
C7WC1	0.46	abcd	1.00	fgh	1.17	a	1.22	c	1.28	b	1.37	b
C7WC2	0.40	abcd	0.87	ij	0.91	bcd	0.99	de	0.94	de	1.05	de
C7WC3	0.39	bcd	0.69	m	0.76	ef	0.89	defg	0.81	fgh	0.76	hi

Notes: C1: IRR 425, C2: IRR 428, C3: IRR 429, C4: IRR 434, C5: IRR 440, C6: RRIC 100, C7: BPM 24, WC1: 30%, WC2: 60%, WC3: 90%. Data is represented by ±SD value. The same letters were not significantly different from each other (P<0.05) using Tukey's test.

The results of total chlorophyll content analysis with the given water content showed a significant effect in all six observations, as depicted in Table 5.

**Figure 4.**
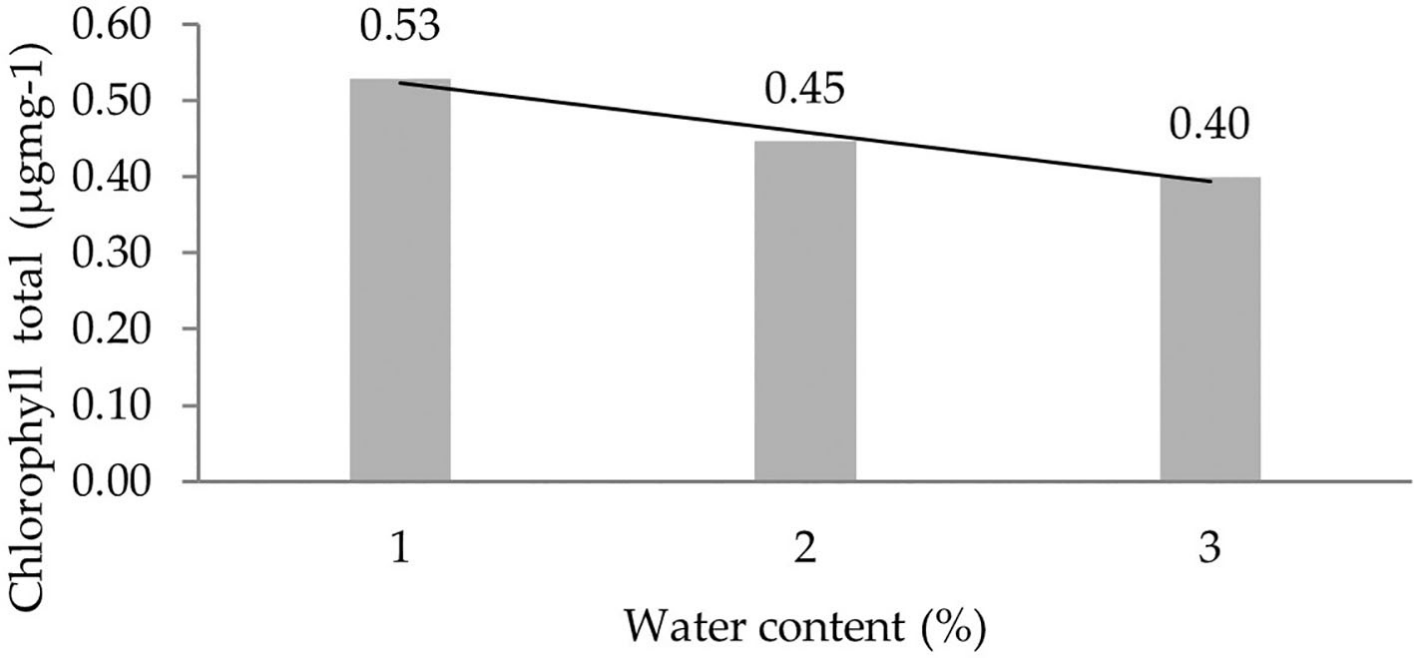
Pattern of total chlorophyll content linear curve as a result of orthogonal polynomial analysis. 1: 30%; 2: 60%; 3: 90%.

The analysis of total chlorophyll content levels due to the interaction between IRR 400 series, RRIC 100, and BPM 24 and water content (30%, 60%, 90%) showed significant differences in all six observations ( Table 5). Orthogonal polynomial regression shows a linear curve, where the water content at 30% has the highest total chlorophyll content value. The linear curve shows that the total chlorophyll content increases with the decreasing water content. The orthogonal polynomial linear curve pattern of total chlorophyll content of several clones of IRR 400 series, RRIC 100, and BPM 24 can be seen in Figure 4.

### Hydrogen peroxidase/H _2_O _2_ (μmolg ^-1^)

The results of total the H _2_O _2_ analysis with different clone types showed a significantly different effect in two of the observations (third and fourth) ( Table 6).

**Table 6.** H _2_O _2_ levels (μmolg ^-1^) in different clones, different water content (%), interactions between clones and water content (%).

Treatments	H _2_O _2_ (μmol g ^-1^)											
	Period to											
	1		2		3		4		5		6	
**Clones (C)**												
IRR 425	0.780	a	0.752	a	0.771	ab	0.746	bc	0.738	a	0.736	a
IRR 428	0.801	a	0.790	a	0.759	ab	0.734	c	0.738	a	0.726	a
IRR 429	0.775	a	0.754	a	0.740	b	0.736	c	0.742	a	0.724	a
IRR 434	0.786	a	0.788	a	0.765	ab	0.777	ab	0.750	a	0.719	a
IRR 440	0.790	a	0.806	a	0.768	ab	0.790	a	0.753	a	0.732	a
RRIC 100	0.782	a	0.761	a	0.790	a	0.785	a	0.759	a	0.728	a
BPM 24	0.798	a	0.749	a	0.771	ab	0.768	abc	0.752	a	0.732	a
**Water content (WC)**												
30%	0.794	a	0.780	a	0.764	a	0.756	a	0.740	a	0.729	a
60%	0.788	a	0.770	a	0.767	a	0.757	a	0.752	a	0.725	a
90%	0.780	a	0.764	a	0.767	a	0.774	a	0.751	a	0.730	a
**Interactions C × WC**												
C1WC1	0.783	a	0.730	a	0.771	a	0.737	abc	0.725	a	0.725	a
C1WC2	0.780	a	0.768	a	0.779	a	0.760	bc	0.737	a	0.740	a
C1WC3	0.777	a	0.758	a	0.764	a	0.742	abc	0.752	a	0.743	a
C2WC1	0.827	a	0.842	a	0.767	a	0.730	bc	0.728	a	0.728	a
C2WC2	0.785	a	0.766	a	0.754	a	0.732	bc	0.748	a	0.731	a
C2WC3	0.790	a	0.762	a	0.756	a	0.740	bc	0.736	a	0.718	a
C3WC1	0.773	a	0.773	a	0.734	a	0.725	c	0.745	a	0.725	a
C3WC2	0.767	a	0.730	a	0.732	a	0.732	bc	0.736	a	0.715	a
C3WC3	0.785	a	0.759	a	0.753	a	0.750	abc	0.746	a	0.730	a
C4WC1	0.782	a	0.798	a	0.768	a	0.780	abc	0.735	a	0.727	a
C4WC2	0.796	a	0.799	a	0.773	a	0.799	abc	0.758	a	0.714	a
C4WC3	0.779	a	0.766	a	0.753	a	0.753	abc	0.756	a	0.716	a
C5WC1	0.812	a	0.837	a	0.780	a	0.804	ab	0.744	a	0.740	a
C5WC2	0.779	a	0.776	a	0.762	a	0.748	a	0.751	a	0.716	a
C5WC3	0.780	a	0.806	a	0.762	a	0.817	abc	0.765	a	0.739	a
C6WC1	0.787	a	0.745	a	0.771	a	0.784	abc	0.769	a	0.728	a
C6WC2	0.793	a	0.792	a	0.807	a	0.776	abc	0.745	a	0.729	a
C6WC3	0.767	a	0.746	a	0.793	a	0.795	abc	0.763	a	0.726	a
C7WC1	0.795	a	0.734	a	0.760	a	0.731	a	0.734	a	0.730	a
C7WC2	0.718	a	0.761	a	0.764	a	0.750	abc	0.786	a	0.728	a
C7WC3	0.780	a	0.752	a	0.790	a	0.823	bc	0.737	a	0.739	a

Notes: C1: IRR 425, C2: IRR 428, C3: IRR 429, C4: IRR 434, C5: IRR 440, C6: RRIC 100, C7: BPM 24, WC1: 30%, WC2: 60%, WC3: 90%. Data is represented by ±SD value. The same letters were not significantly different from each other (P<0.05) using Tukey's test.

The results of H _2_O _2_ analysis at different water content levels did not show significant differences in any of the observations ( Table 6).

The analysis of H _2_O _2_ levels (μmol g ^-1^) due to interactions between IRR 400 series, RRIC 100, and BPM 24 and given water content (30%, 60%, 90%) showed a significantly different effect in just one observation (fourth) ( Table 6).

**Figure 5.**
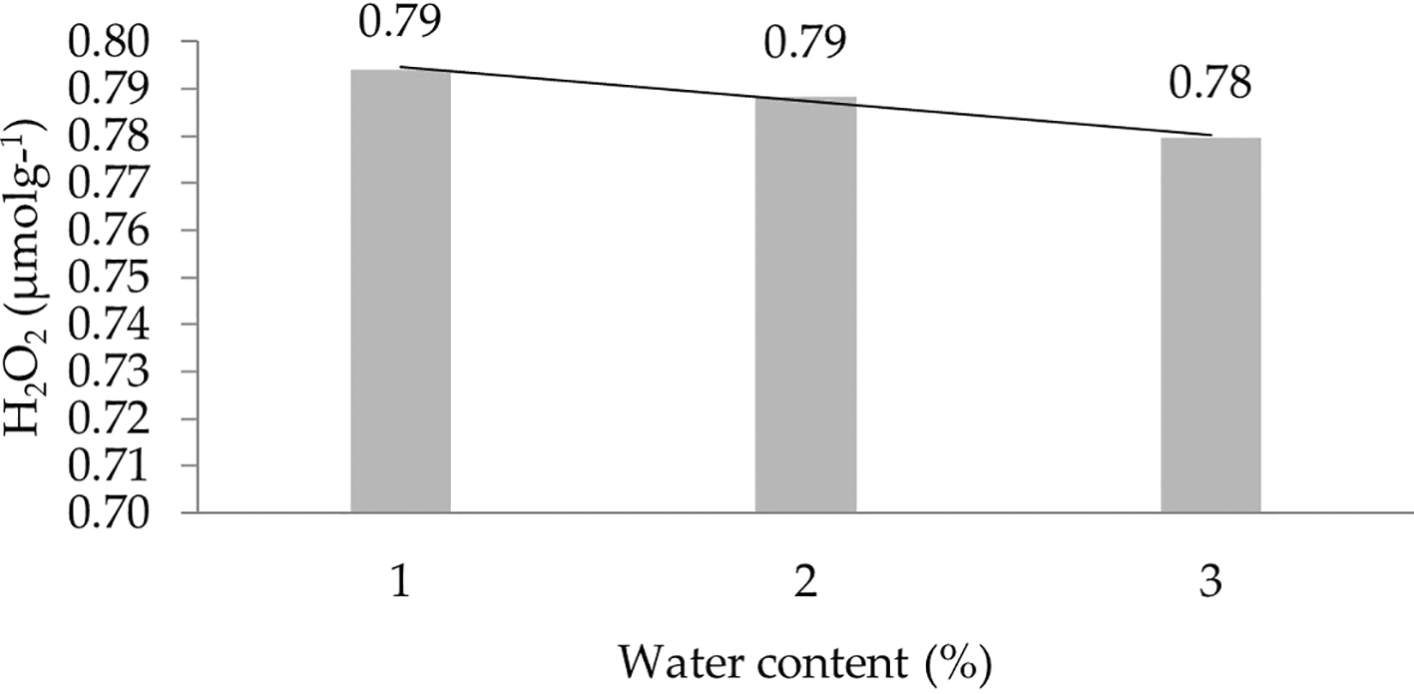
Pattern of H _2_O _2_ (μmol g ^-1^) linear curve as a result of orthogonal polynomial analysis. 1: 30%; 2: 60%; 3: 90%.

The effect of water content on the H _2_O _2_ characteristic shows a linear regression curve based on orthogonal polynomials. This indicates that the lower the water content, the higher the concentration of H _2_O _2_. The linear regression pattern between H _2_O _2_ content and water content can be seen in Figure 5.

### Ascorbate peroxidase/APX (units mg ^-1^)

The results of APX analysis with different clone types were not significantly different in any of the observations ( Table 7).

**Table 7.** APX levels (units mg ^-1^) in different clones, different water content (%) and interactions between clones and water content (%).

Treatments	APX level (units mg ^-1^)											
	Period to											
	1		2		3		4		5		6	
**Clones (C)**												
IRR 425	1.48	a	1.13	a	1.01	a	1.21	a	1.29	a	1.14	a
IRR 428	1.37	a	1.20	a	1.06	a	1.32	a	1.41	a	1.03	a
IRR 429	1.25	a	1.32	a	1.01	a	1.23	a	1.41	a	1.11	a
IRR 434	1.30	a	1.29	a	1.05	a	1.44	a	1.40	a	0.96	a
IRR 440	1.27	a	1.29	a	1.15	a	1.36	a	1.34	a	1.12	a
RRIC 100	1.22	a	1.23	a	1.10	a	1.33	a	1.33	a	1.02	a
BPM 24	1.31	a	1.23	a	1.00	a	1.27	a	1.24	a	0.99	a
**Water content (WC)**												
30%	1.38	a	1.15	a	1.05	a	1.27	a	1.34	a	1.05	a
60%	1.32	a	1.34	a	1.07	a	1.35	a	1.38	a	1.03	a
90%	1.24	a	1.24	a	1.05	a	1.31	a	1.33	a	1.09	a
**Interactions C × WC**												
C1WC1	1.58	a	1.18	a	1.03	a	1.03	a	1.23	a	1.17	a
C1WC2	1.42	a	1.24	a	0.95	a	1.25	a	1.27	a	1.08	a
C1WC3	1.44	a	0.98	a	1.05	a	1.36	a	1.36	a	1.17	a
C2WC1	1.47	a	1.08	a	1.04	a	1.45	a	1.55	a	1.08	a
C2WC2	1.53	a	1.20	a	1.10	a	1.31	a	1.27	a	1.04	a
C2WC3	1.10	a	1.30	a	1.06	a	1.20	a	1.42	a	0.97	a
C3WC1	1.38	a	1.23	a	1.01	a	1.22	a	1.43	a	1.08	a
C3WC2	1.25	a	1.35	a	0.91	a	1.40	a	1.45	a	1.11	a
C3WC3	1.12	a	1.37	a	1.11	a	1.08	a	1.36	a	1.15	a
C4WC1	1.45	a	1.16	a	0.98	a	1.59	a	1.34	a	0.96	a
C4WC2	1.25	a	1.46	a	1.13	a	1.32	a	1.56	a	1.00	a
C4WC3	1.21	a	1.24	a	1.04	a	1.42	a	1.32	a	0.93	a
C5WC1	1.38	a	1.22	a	1.09	a	1.31	a	1.12	a	1.06	a
C5WC2	1.17	a	1.40	a	1.23	a	1.36	a	1.41	a	1.05	a
C5WC3	1.26	a	1.26	a	1.15	a	1.42	a	1.48	a	1.25	a
C6WC1	1.15	a	1.06	a	1.11	a	1.18	a	1.52	a	1.01	a
C6WC2	1.26	a	1.46	a	1.23	a	1.45	a	1.37	a	0.91	a
C6WC3	1.25	a	1.17	a	0.97	a	1.35	a	1.10	a	1.15	a
C7WC1	1.24	a	1.11	a	1.06	a	1.14	a	1.16	a	0.97	a
C7WC2	1.38	a	1.23	a	0.95	a	1.37	a	1.30	a	0.98	a
C7WC3	1.30	a	1.35	a	1.00	a	1.32	a	1.26	a	1.01	a

Notes: C1: IRR 425, C2: IRR 428, C3: IRR 429, C4: IRR 434, C5: IRR 440, C6: RRIC 100, C7: BPM 24, WC1: 30%, WC2: 60%, WC3: 90%. Data is represented by ±SD value. The same letters were not significantly different from each other (P<0.05) using Tukey's test.

The analysis results of APX at different water content levels were not significantly different in any of the observations ( Table 7).

The analysis of APX levels (units mg ^-1^) due to the interaction between IRR 400 series, RRIC 100, and BPM 24 and water content (30%, 60%, 90%) did not show any significant differences in any of the observations ( Table 7).

### Superoxide Dismutase/SOD (units mg ^-1^)

The SOD analysis with clone types showed a significant difference in three of the observations ( Table 8).

**Table 8.** SOD levels (units mg ^-1^) in different clones, different water content (%) and interactions between clones and water content (%).

Treatments	SOD (units mg ^-1^)											
	Period to											
	1		2		3		4		5		6	
**Clones (C)**												
IRR 425	2.43	a	2.56	bc	2.28	a	2.29	a	2.39	a	2.42	bc
IRR 428	2.36	ab	2.57	abc	2.25	a	2.29	a	2.39	a	2.45	bc
IRR 429	2.23	c	2.60	ab	2.21	a	2.33	a	2.44	a	2.44	bc
IRR 434	2.38	a	2.54	bc	2.27	a	2.23	a	2.44	a	2.49	bc
IRR 440	2.34	abc	2.70	a	2.29	a	2.32	a	2.38	a	2.40	c
RRIC 100	2.27	bc	2.44	c	2.28	a	2.29	a	2.34	a	2.62	a
BPM 24	2.32	bc	2.52	bc	2.21	a	2.35	a	2.43	a	2.52	ab
**Water content (WC)**												
30%	2.28	b	2.56	a	2.23	a	2.32	a	2.39	ab	2.49	a
60%	2.42	a	2.53	a	2.26	a	2.28	a	2.45	a	2.43	b
90%	2.30	b	2.59	a	2.27	a	2.30	a	2.36	b	2.52	a
**Interaction C × WC**												
C1W1	2.29	cdefg	2.45	ab	2.24	a	2.43	a	2.36	ab	2.37	fgh
C1W2	2.35	bcdefg	2.60	ab	2.37	a	2.20	a	2.44	ab	2.35	efgh
C1W3	2.63	a	2.62	ab	2.22	a	2.23	a	2.36	ab	2.54	abcdef
C2W1	2.27	cdefg	2.61	ab	2.24	a	2.35	a	2.32	ab	2.37	defgh
C2W2	2.52	abc	2.54	ab	2.24	a	2.29	a	2.59	a	2.38	efgh
C2W3	2.30	defg	2.56	ab	2.26	a	2.23	a	2.28	b	2.61	abcd
C3W1	2.29	cdefg	2.59	ab	2.19	a	2.36	a	2.40	ab	2.44	cdefgh
C3W2	2.24	cdefg	2.54	ab	2.20	a	2.25	a	2.45	ab	2.42	bcdefgh
C3W3	2.17	g	2.67	ab	2.24	a	2.38	a	2.47	ab	2.45	cdefgh
C4W1	2.43	abcd	2.61	ab	2.19	a	2.20	a	2.35	ab	2.66	ab
C4W2	2.49	abcd	2.44	b	2.25	a	2.31	a	2.51	ab	2.36	bcdefgh
C4W3	2.24	efg	2.57	ab	2.36	a	2.20	a	2.46	b	2.45	fgh
C5W1	2.26	defg	2.69	ab	2.39	a	2.29	a	2.42	ab	2.28	gh
C5W2	2.56	ab	2.67	ab	2.25	a	2.31	a	2.44	ab	2.31	h
C5W3	2.19	fg	2.73	b	2.22	a	2.37	a	2.27	b	2.63	abcd
C6W1	2.20	efg	2.46	ab	2.26	a	2.29	a	2.41	ab	2.59	abcde
C6W2	2.40	abcdef	2.44	ab	2.31	a	2.27	a	2.35	b	2.64	abcd
C6W3	2.21	efg	2.43	b	2.27	a	2.31	a	2.27	b	2.61	abcd
C7W1	2.25	efg	2.49	ab	2.12	a	2.33	a	2.47	ab	2.69	a
C7W2	2.35	bcdefg	2.51	ab	2.20	a	2.35	a	2.39	ab	2.53	abcdefg
C7W3	2.36	bcdefg	2.57	ab	2.30	a	2.37	a	2.42	ab	2.33	fgh

Notes: C1: IRR 425, C2: IRR 428, C3: IRR 429, C4: IRR 434, C5: IRR 440, C6: RRIC 100, C7: BPM 24, WC1: 30%, WC2: 60%, WC3: 90%. Data is represented by ±SD value. The same letters were not significantly different from each other (P<0.05) using Tukey's test.

Analysis of SOD (units mg ^-1^) levels at different water content levels showed significant differences in three of the observations, as depicted in Table 8.

The analysis of SOD levels due to interaction between IRR 400 series, RRIC 100, and BPM 24 and water content (30%, 60%, 90%) showed significant differences in two observations ( Table 8).

### Peroxide dismutase/POD (units mg ^-1^)

The POD analysis with different types of clones showed significant differences in two observations, as shown in Table 9.

**Table 9.** POD levels (units mg ^-1^) in different clones, different water content (%), interactions between clones and water content (%).

Treatments	POD (units mg ^-1^)											
	Period to											
	1		2		3		4		5		6	
**Clones (C)**												
IRR 425	0.869	a	0.903	a	0.856	a	0.932	a	0.918	a	0.858	a
IRR 428	0.898	a	0.940	a	0.850	a	0.919	a	0.923	a	0.870	a
IRR 429	0.887	a	0.895	a	0.875	a	0.914	a	0.873	a	0.834	a
IRR 434	0.891	a	0.906	a	0.881	a	0.846	b	0.919	a	0.848	a
IRR 440	0.889	a	0.901	a	0.870	a	0.918	a	0.917	a	0.853	a
RRIC 100	0.881	a	0.904	a	0.898	a	0.907	ab	0.923	a	0.829	a
BPM 24	0.891	a	0.924	a	0.874	a	0.938	a	0.923	a	0.875	a
**Water content (WC)**												
30%	0.874	b	0.912	a	0.874	a	0.912	a	0.919	a	0.864	a
60%	0.895	a	0.915	a	0.875	a	0.906	a	0.913	a	0.862	a
90%	0.891	a	0.904	a	0.867	a	0.914	a	0.909	a	0.831	a
**Interaction C × WC**												
C1W1	0.869	cdef	0.919	a	0.891	a	0.943	a	0.961	a	0.859	a
C1W2	0.813	fgh	0.931	a	0.867	a	0.940	a	0.900	a	0.836	a
C1W3	0.926	fgh	0.860	a	0.810	a	0.915	ab	0.893	a	0.879	a
C2W1	0.901	bcdef	0.939	a	0.803	a	0.918	ab	0.942	a	0.875	a
C2W2	0.919	abc	0.921	a	0.854	a	0.899	ab	0.937	a	0.899	a
C2W3	0.874	bcd	0.961	a	0.893	a	0.940	a	0.889	a	0.836	a
C3W1	0.826	abc	0.897	a	0.855	a	0.915	b	0.903	a	0.835	a
C3W2	0.897	abc	0.882	a	0.912	a	0.930	ab	0.857	a	0.855	a
C3W3	0.938	ab	0.905	a	0.857	a	0.899	ab	0.858	a	0.811	a
C4W1	0.917	abc	0.897	a	0.920	a	0.784	ab	0.854	a	0.900	a
C4W2	0.920	abcd	0.917	a	0.795	a	0.860	ab	0.936	a	0.849	a
C4W3	0.836	ab	0.905	a	0.929	a	0.894	a	0.967	a	0.795	a
C5W1	0.774	h	0.894	a	0.852	a	0.909	ab	0.929	a	0.879	a
C5W2	0.917	abcd	0.904	a	0.878	a	0.915	ab	0.923	a	0.842	a
C5W3	0.974	a	0.904	a	0.879	a	0.929	a	0.900	a	0.837	a
C6W1	0.908	bcd	0.931	a	0.899	a	0.954	a	0.938	a	0.805	a
C6W2	0.944	abcd	0.923	a	0.904	a	0.863	ab	0.900	a	0.872	a
C6W3	0.790	gh	0.858	a	0.890	a	0.902	ab	0.930	a	0.809	a
C7W1	0.922	abc	0.905	a	0.896	a	0.961	a	0.907	a	0.899	a
C7W2	0.854	bcde	0.931	a	0.913	a	0.935	a	0.936	a	0.897	a
C7W3	0.897	defg	0.937	a	0.814	a	0.919	ab	0.925	a	0.848	a

Notes: C1: IRR 425, C2: IRR 428, C3: IRR 429, C4: IRR 434, C5: IRR 440, C6: RRIC 100, C7: BPM 24, WC1: 30%, WC2: 60%, WC3: 90%. Data is represented by ±SD value. The same letters were not significantly different from each other (P< 0.05) using Tukey's test.

The analysis of POD levels at different water content levels showed a significant difference in one observation, as depicted in Table 9.

The analysis of POD levels due to interaction between IRR 400 series, RRIC 100, and BPM 24 and given water content (30%, 60%, 90%) showed a significant difference in just one observation ( Table 9).

## Discussion

Plants respond and adapt to the change in environmental conditions such as drought stress by altering its physiological and biochemical activities to overcome such stressful situation. ^9^ ^,^ ^10^

The plants respond to the environmental stress by altering its physiological activities so as to maintain its growth and development without succumbing to the strees by producing compounds that render tolerance. The accumulation of osmoprotectants is a key biochemical property in plants tolerant to abiotic stress, ^10^ ^,^ ^21^ and there is clear evidence that osmotic adjustment sustains crop yields under drought stress. ^9^ Drought stress causes changes in amino acid metabolism. The accumulated solutes protect cellular proteins, organelles, membranes, and various enzymes against drought stress.

Several physiological characteristics were analyzed to see the effect of water content on IRR 400 series, RRIC 100, and BPM 24 rubber clones. Some of the dissolved substances assessed in this study were total sugar, proline, and chlorophyll (a, b, total). The correlation of total sugar content to each clone showed different effects. Each clone had altered levels of total sugar content under stress conditions. The RRIC 100 is a drought tolerant clone in the field. The increase in total sugar content was seen in most observations of the water content treatment. The interaction between clone type and water content can increase total sugar content, especially when the water content added is 30%. Initial hypotheses suggest that each clone has the ability to adapt to water shortages. The accumulation of soluble sugars in plant cells subjected to drought stress is responsible for the osmotic adjustment. ^25^ Sugar accumulation in drought-stressed plants is controlled by several mechanisms that affect soluble sugar formation and transfer in leaves. ^26^ Similar results of increased total sugar accumulation have been observed in drought-stressed soybeans ^26^ and sugarcane. ^27^

This study showed different proline values among the tested clones. All six observations indicate that clones have the ability to survive drought. The IRR 425 clone had the highest proline levels in three observations. Meanwhile, the IRR 440 had the lowest proline levels in four observations. Assessing the proline characteristic, the initial assumption was that IRR 425 had a stronger adaptation compared with other clones, especially the IRR 440. Regarding different water contents (30%, 60%, 90%), the proline levels at 30% were greater than at 60% and 90%. This indicated that a higher amount of proline accumulated when the water content was lower in the growth medium. Proline is an important amino acid as it is an osmotic-compatible molecule and has the potential to form a defense system to increase drought tolerance. Proline acts as an antioxidative defense molecule and causes stress signaling. ^12^ It is classified as an osmoprotectant, as it increases hyperosmolarity and antioxidant enzymes’ activity. ^28^ Increased proline content in drought-stress plants can maintain turgor for continued growth. ^29^ Hence, proline accumulation correlates with osmoprotection. ^30^ The interaction between clone types and moisture content indicated that each clone showed a different effect in all six observations. The clones had high proline levels when treated with 30% water content. This shows that the clonal factor still has to be planted in the field which is classified as a drought environment. The proline content has been shown to increase about 10-fold in mungbean, ^31^ maize, ^32^ millet, ^12^ ^,^ ^33^ ^,^ ^34^ nyamplung, ^35^ and soybean ^26^ under drought stress.

Chlorophyll is the main pigment found in chloroplasts. ^36^ The three main functions of chlorophyll in the photosynthesis process are harnessing solar energy, triggering CO _2_ fixation to produce carbohydrates, and providing energy for the ecosystem as a whole. Chlorophyll a and chlorophyll b absorb the most light in the red part (600–700 nm) and absorb the least in the green part (500–600 nm). ^35^ ^–^ ^37^ In this study, it was seen that chlorophyll a, b, and total levels at 30% were higher than at 90%. The genotype BC678 and BC404 of corn cultivars showed resistant to drought stress have highest chlorophyll index and higher potential yield. ^38^

This is presumably because the rubber plant is a perennial plant that is able to adapt when water conditions are on a small scale due to the root structure of the rubber plant. It is a bud-grafted clonal material (of each varuet/genotype) which does not have a tap root to mine water from the deeper soil. In addition, when stressed, the lateral roots will grow even more to take advantage of the existing water on the surface. ^39^

This study was conducted in greenhouse conditions where the water provided to the plants will not be lost as water treated gradually. Even though the polybags made small holes to drain excess water during its pre-treatment conditions. Besides that, the polybag surface is also covered by plastic to minimize the occurrence of evapotranspiration from the planting medium. Another factor that can be used as a cause of high chlorophyll levels is ability to adjust osmolyte content (sugar, proline, glycine betaine, ABA, ethylene, and others), and the nature of the rubber plant itself (plants that will shed leaves naturally every year).

Antioxidants are active substances that naturally detoxify free radicals (ROS). The presence of oxidative stress and an abundance of antioxidants are important activities for metabolic protection when plants are under stress. ROS in the form of free radicals and peroxides are molecules derived from oxygen metabolism. The toxic effects of ROS can be countered by antioxidant enzymatic as well as non-enzymatic systems, such as SOD, CAT, APX, GR, ascorbic acid (AsA), tocopherols, glutathione and phenolic compounds, and others. Typically, each cellular compartment contains more than one enzymatic activity that detoxifies ROS. The presence of these enzymes in almost all cells plays an important role in ROS detoxification for plant survival. ^40^

H _2_O _2_ has several important roles in various biochemical and physiological processes. Long plant life and long growth processes result in H _2_O _2_ crossing cellular membranes and potentially acting as a signal in the signal transduction pathway of stress. This pathway triggers various responses of the adaptation process in the environment where the plant is cultivated. ^41^ High levels of H _2_O _2_ cause oxidative stress, which then causes cell damage and death. ^42^ However, optimal levels of H _2_O _2_ can increase tolerance to abiotic stresses through modulation of various physiological processes, including photosynthesis, opening and closing of stomata, osmotic adjustment, and ROS detoxification. ^41^ ^,^ ^42^ ROS detoxification is very important in maintaining the structural and membrane integrity of cellular organelles and keeping them fully functional under stress. The accumulation of optimal amounts of H _2_O _2_ triggers the occurrence of chitinase proteins that can produce calcium homeostasis, ion channels, phosphatases, transcription factors, and abscisic acid (ABA), signaling responses to stress. ^43^

APX in ascorbate–glutathione (AsA–GSH) cycling enzymes is responsible for the decomposition of H _2_O _2_ produced by SOD in different cellular organelles. APX plays a key role in both drought stress response and recovery after drought. ^43^ ^,^ ^44^ APX is an integral component of the (ASC–GSH) cycle. APX performs the same function in the cytosol and chloroplasts. APX reduces H _2_O _2_ to H _2_O and docosahexaenoic acid (DHA), using AsA as a reducing agent.

$$H_{2}O_{2}+\mathrm{AA}\to {\;}_{2}H_{2}O+\mathrm{DHA}$$

The APX family consists of five isoforms based on different sites of amino acid formation, such as the cytosol, mitochondria, peroxisomes, and chloroplastids (stroma and thylakoids). ^45^ APX is widely distributed and has a better affinity to H _2_O _2_, especially in terms of more efficient uptake of H _2_O _2_ in times of stress. ^45^ ^–^ ^48^

Though the SOD levels in each clone showed a significant effect due to water content, it was limited to a few observations because drought affects the metabolic activity of clones. Likewise for the levels of SOD at a given water content. Water content of 30% showed relatively the same SOD activity as 60% and 90% in all observations. This indicates that SOD were formed in low levels in the observations and therefore cannot be used as a marker of tolerance for these tested clones. SOD is one of the key components of cell protection against oxidative stress. The SOD has three different isoenzymes distributed between organelles. Cu/Zn-SOD is predominantly located in the chloroplasts, cytosol, and peroxisomes, whereas FeSOD and MnSOD are mostly found in chloroplasts and mitochondria, respectively. ^49^ POD and SOD activities increased sharply in rubber seedlings after being subject to drought stress. This suggests that the photosynthetic activity and lipid integrity of the cell membranes are rapidly attenuated by drought stress. SODs are metalloenzymes that play an important role in ROS reactions, or, in other words, are able to neutralize the negative effects of ROS. The decrease in substrate binding affinity to SOD as well as a decrease in one isozyme band of SOD under drought conditions may be responsible for the resistance. Plants that have a higher induced SOD activity show more tolerance to abiotic stresses. Numerous studies have shown that plants are able to better eliminate the negative effects of ROS produced under stressful situations when their SOD activity is higher, provided there are more SOD isoenzymes present.

POD had low values in all six observations of some clones. The low POD indicated that the effect of some water content percentages given during the six observations on several different clones did not have a significant effect. This indicates that the POD characteristics cannot be used as a reference for plant tolerance to drought stress. Plants that produce more POD under conditions of drought stress will be able to survive by eliminating the effects of ROS. In general, the activity of POD and other antioxidant enzymes will automatically have a higher value in tolerant clones/varieties and will have a lower value in susceptible clones/varieties. This indicates that drought-tolerant clones/varieties will be more efficient in removing H _2_O _2_ to produce optimal protection. The tolerance of some genotypes to environmental stresses has been associated with higher antioxidant enzyme activity. Drought-tolerant species of pigeon pea ( *Cajanus cajan*), ^50^ wheat ( *Triticum aestivum*), ^51^ ^,^ ^52^ and black bean ( *Phaseolus mungo*) ^49^ have higher SOD, POD, and CAT activities than drought-sensitive species. The results of this study indicate that ROS enzymes, which play a crucial role in the drought-tolerance mechanism under the drought treatment, have been identified in clones IRR 425, IRR 428, IRR 429, IRR 434, IRR 440, RRIC 100, and BPM 24. Based on the findings, several analyses have been carried out on physiological characteristics to determine the effects of water content on a greenhouse scale.

## Conclusions

Based on the physiological and biochemical parameters made in this investigation to determine the effect of water content in a greenhouse scale, the findings indicate that only few parameters are reliable as indicators of drought stress tolerance. For e.g. the parameters such as total sugar content and proline were exhibiting strong correlation with drought stress tolerance in the IRR 400 series. They were produced at higher levels in plants treated with lesser water content. However, the parameters such as chlorophyll a, b, and total, H _2_O _2_, APX SOD, and POD did not show any correlation to use as drought tolerance indicators in this experiment.

## Data availability

### Underlying data

This project contains the following underlying data:

Figshare: Data set: Physiological Characters of IRR 400 Series Rubber Clones ( *Heveabrasiliensis*Muell. Arg.) on Drought Stress. https://doi.org/10.6084/m9.figshare.21708230.v2 ^52^

• Biochemistry characters.xlsx (datasets for total sugar, chlorophyll (a, b, total), proline, H _2_O _2_, APX, SOD, POD)

Figshare: Data set: Physiological Characters of IRR 400 Series Rubber Clones ( *Heveabrasiliensis*Muell. Arg.) on Drought Stress. https://doi.org/10.6084/m9.figshare.21708275.v2 ^53^

• Polyortogonal_test.xlsx (datasets for total sugar, chlorophyll (a, b, total), proline, H _2_O _2_, APX, SOD, POD)

### Extended data

Figshare: Data set: Physiological Characters of IRR 400 Series Rubber Clones ( *Heveabrasiliensis*Muell. Arg.) on Drought Stress. https://doi.org/10.6084/m9.figshare.21645116.v5 ^54^

This project contains the following extended data:

Data are available under the terms of the Creative Commons Attribution 4.0 International license (CC-BY 4.0).
