# Supplementary material for: Physiological characteristics of IRR 400 series rubber clones ( Hevea brasiliensis Muell. Arg.) under drought stress
Source: F1000Res. 2023 Dec 4;12:106. Originally published 2023 Jan 27. [Version 4] doi: 10.12688/f1000research.129421.4 (PMC11809680; doi:10.12688/f1000research.129421.4)
Supplement: Peer review history

# Reviewer report: Reviewer response for version 4

**DOI:** 10.5256/f1000research.159406.r350669

**Published:** 2025-01-02

**Authors:** Boon Chin Tan

**Affiliations:** 1 Universiti Malaya, Kuala Lumpur, Malaysia

The manuscript aims to identify drought-tolerant traits to select drought-tolerant rubber clones at the juvenile stage. Overall, the study is conducted in an acceptable manner, with appropriate methodologies for achieving the objectives. The authors have also revised and improved the manuscript accordingly. However, the manuscript lacks sufficient detail in the methodology section, requiring some clarifications and improvements.

Methods

 The work was conducted using 6–12-month-old plants. Could this age range introduce variability that may affect comparisons between different clones?

Experimental design

 How did the authors implement drought treatment and achieve 30%, 60%, and 90% soil water content levels?

 Did the authors measure soil water content? If so, which apparatus was used?

 Were any fertilizers applied during the treatment?

 Where were the tested clones sourced from?

 The term “normal leaves” should be revised to “disease-free leaves.”

 “Six months observation” should be changed to “six months' observation”.

Results

 The sentence “the highest total sugar content four times, and the IRR 425 clone had the lowest four times” needs revision for clarity, particularly the meaning of "four times."

 APX, SOD, and POD (units mg-1): The authors should define the “units” for each assay.

Tables

 The unit for “period to” should be specified as “month.”

 It is necessary to mention in the methodology that samples were harvested monthly.

 The data analysis method should be explained, and the meaning of the letters should be defined in the table footnotes.

Conclusion

 “For e.g.” should be changed to “For example”.

Is the work clearly and accurately presented and does it cite the current literature?

Yes

If applicable, is the statistical analysis and its interpretation appropriate?

I cannot comment. A qualified statistician is required.

Are all the source data underlying the results available to ensure full reproducibility?

Partly

Is the study design appropriate and is the work technically sound?

Yes

Are the conclusions drawn adequately supported by the results?

Yes

Are sufficient details of methods and analysis provided to allow replication by others?

Partly

Reviewer Expertise:

Plant biotechnology and physiology

I confirm that I have read this submission and believe that I have an appropriate level of expertise to confirm that it is of an acceptable scientific standard, however I have significant reservations, as outlined above.

# Reviewer report: Reviewer response for version 3

**DOI:** 10.5256/f1000research.157145.r214777

**Published:** 2023-11-07

**Authors:** Mohamed sathik Thirruvithamkottil

**Affiliations:** 1 Rubber Research Institute of India, Rubber Board, Kottayam, Kerala, India

1. Introduction 2nd paragraph,

 One of the primary sources of natural rubber **is **producing plants - here **is** is not required.

 2. signaling pathways and no comma mark **(,) **is needed  before ‘and’

 3. In times of drought stress, lack of sufficient water combined with the increased CO _2_ in the atmosphere can cause plant death. ^13^

 I could not see the death in the reference mentioned above.  Please correct it or omit the point as it does not look relevant.

 4. The present study **is** aimed (is missing)…… to obtain character selection is not the appropriate way of communication… should have been ‘to identify drought tolerant traits in order to select or identify drought-tolerant clones at juvenile stage itself.

 Discussion

 1. The previous point mentioned below is not addressed

 Second paragraph, fourth line says’ RRIC 100 is a **dry tolerant clone in the field**’.  Does the author mean that it is drought tolerant because both are different.  Drying is desiccation where as drought is a complete water deficit stress which is different from drying alone.  Hence, utmost care should have been taken while drafting the manuscript.

**2. Increased proline content in drought-stress plants can provide high energy to increase plant growth in water-deficit conditions. ^29^**

 The above statement as per ref no. 29 was verified and in none of the chapters it says that proline can provide high energy. **Please clarify**

 3. This is presumably because the rubber plant is a perennial plant that is able to adapt when water conditions are on a small scale due to the root structure of the rubber plant. It has a taproot that will grow even deeper to find water farther from the ground. 

**In my previous comment, I said that it is a bud-grafted clonal material (of each varuet/genotype) which does not have a tap root to mine water from the deeper soil as mentioned in the above sentence.  Please check or justify.**

**4. **From the same paragraph, for the first time we can see mentioning of polybags where it is also mentioned that growing media (that means is it not soil?) and no hole in the polybags (which are generally made with holes to drain excess water during its pre-treatment conditions.

**Please justify!**

**5. **The decrease in substrate binding affinity to SOD as well as a decrease in one isozyme band of SOD under drought conditions may be responsible for the resistance.

**Please justify how the decrease in subtrate binding affinity (to SOD) can impart resistance?**

**Conclusion**

 However, the parameters such as chlorophyll a, b, and total, H _2_O _2_, APX SOD, and POD did not show any correlation to **used **as drought tolerance indicators in this experiment

**"Used'** should be changed to ' **use'**

Is the work clearly and accurately presented and does it cite the current literature?

Partly

If applicable, is the statistical analysis and its interpretation appropriate?

No

Are all the source data underlying the results available to ensure full reproducibility?

Partly

Is the study design appropriate and is the work technically sound?

Yes

Are the conclusions drawn adequately supported by the results?

Partly

Are sufficient details of methods and analysis provided to allow replication by others?

Partly

Reviewer Expertise:

Plant Physiology, Molecular Biology, Plant Breeding, etc.

I confirm that I have read this submission and believe that I have an appropriate level of expertise to confirm that it is of an acceptable scientific standard, however I have significant reservations, as outlined above.

# Response

**Published:** 2023-11-26

**Authors:** Mohammad Basyuni

**Affiliations:** Universitas Sumatera Utara, Indonesia

**We thank Reviewer 1 for his comments on the merit of our work and for providing constructive comments and suggestions to improve our manuscript. We have revised the manuscript by addressing suggestions as follows:**

 APPROVED WITH RESERVATIONS

 info_outline

 1. Introduction 2nd paragraph,

 One of the primary sources of natural rubber is producing plants - here is is not required.

**Is has been deleted**

 2. signaling pathways and no comma mark (,) is needed  before ‘and’

**Comma mark (,) has been removed.**

 3. In times of drought stress, lack of sufficient water combined with the increased CO 2 in the atmosphere can cause plant death.13

**The sentence has been corrected to read: The combined rise in atmospheric CO2 concentrations and global air tempera** **ture** **may significantly alter plant water use.**

 I could not see the death in the reference mentioned above.  Please correct it or omit the point as it does not look relevant.

 4. The present study is aimed (is missing)…… to obtain character selection is not the appropriate way of communication… should have been ‘to identify drought tolerant traits in order to select or identify drought-tolerant clones at juvenile stage itself.

**The aim of study has been corrected.**

 Discussion

 1. The previous point mentioned below is not addressed

 Second paragraph, fourth line says’ RRIC 100 is a dry tolerant clone in the field’.  Does the author mean that it is drought tolerant because both are different.  Drying is desiccation where as drought is a complete water deficit stress which is different from drying alone.  Hence, utmost care should have been taken while drafting the manuscript.

**Thank you for the correction. Drying has been revised to drought.**

 2. Increased proline content in drought-stress plants can provide high energy to increase plant growth in water-deficit conditions.

**Thank you for your correction. The sentence has been improved to read: Increased proline content in drought-stress plants can maintain turgor for continued growth.**

 The above statement as per ref no. was verified and in none of the chapters it says that proline can provide high energy.  Please clarify

 3. This is presumably because the rubber plant is a perennial plant that is able to adapt when water conditions are on a small scale due to the root structure of the rubber plant. It has a taproot that will grow even deeper to find water farther from the ground.

 In my previous comment, I said that it is a bud-grafted clonal material (of each varuet/genotype) which does not have a tap root to mine water from the deeper soil as mentioned in the above sentence.  Please check or justify.

**The sentence has been revised to meet Reviewer’s suggestions. Done as recommended.**

 4. From the same paragraph, for the first time we can see mentioning of polybags where it is also mentioned that growing media (that means is it not soil?) and no hole in the polybags (which are generally made with holes to drain excess water during its pre-treatment conditions.

 Please justify!

**We have corrected the sentence to icorporate Reviewer’s comments. Please refer to revised Discussion.**

 5. The decrease in substrate binding affinity to SOD as well as a decrease in one isozyme band of SOD under drought conditions may be responsible for the resistance.

 Please justify how the decrease in subtrate binding affinity (to SOD) can impart resistance?

**This sentence has been deleted to avoid the obscurity to SOD**

 Conclusion

 However, the parameters such as chlorophyll a, b, and total, H2O2, APX SOD, and POD did not show any correlation to used as drought tolerance indicators in this experiment

 "Used' should be changed to 'use'

**“used’ has been revised to ‘use’**

# Reviewer report: Reviewer response for version 2

**DOI:** 10.5256/f1000research.152374.r188303

**Published:** 2023-09-19

**Authors:** Mohamed sathik Thirruvithamkottil

**Affiliations:** 1 Rubber Research Institute of India, Rubber Board, Kottayam, Kerala, India

Introduction second para

 1. One of the primary sources of natural rubber is - please insert with 'producing plants' was found in the Amazon..

 2. northeastern states of India - does not experience drought, instead say the 'Central India'.

Introduction third para

 3. "Based on bio-informatics, there are 20 proteins related to drought stress in rubber..." This can be written as, 'Pasaribu *et al.,* reported...20 drought stress related proteins...', otherwise it would mean that there are only 20 drought related proteins found in Hevea.

Methods, Stages of growth and development.....

 1. second line: insert space between "...Corynespora and"

 2. Change the sentence to 'The leaves were taken between 8.00 AM and 9.00 AM'. Morning is not needed as AM is given.

 3. Data Analysis: First line - change to 'with an interval of three weeks'.

Results

 1. Proline unit mgg ^-1^ should be changed to mg g ^-1^. There should be space between the mg and g. Replace wherever it is applicable as this is found throughout the manuscript.

 2. 'chlorophyll (microgram) ug mg ^-1^'

 3. Instead of "Chlorophyll total change" it to 'Total Chlorophyll content'

 4. Ascorbate peroxidase/APX (units mg ^-1^) - space inserted.

 5. SOD. POD - also (units mg ^-1^)

Discussion - First Paragraph: Please recast it as it does not convey the message properly.

 For e.g. You can try like below.

 'Plants respond and adapt to the change in environmental conditions such as drought stress by altering its physiological and biochemical activities to overcome such stressful situations.'

 The second and third line are mere repetitions of the first line which you can avoid.

Second para: the first line does not convey the message properly.

 I think it should have been - 'The plants respond to the environmental stress by altering its physiological activities so as to maintain its growth and development without succumbing to the stress by producing compounds that render tolerance.'

In third para, third line:

 "Each clone showed its ability to produce total sugar content when stressed." - what do you mean by this. It is obvious that even without stress, total sugar content will always be there. Do you want to say that 'Each clone had altered levels of total sugar content under stress conditions?

Fourth para, third line from the bottom:

 "This shows that the clonal factor still has to be tested in other environments against drought stress." - What do you mean by this sentence? How do you want to test in other environments against drought stress?

6th para...

 "This is presumably because the rubber plant is **an** perennial plant..." - 'an' is not required, instead just use 'a' only.

second line

 "It has a taproot that will grow even deeper to find water farther from the ground." - Generally the bud-grafted plants do not have tap root that can go deeper to find water. The root system will be to a length of about one meter only in the ground below. It only uses the lateral roots to find water on the top layer. So how will you substantiate your statement?

7th para:

 "The condition of this research is a scale research greenhouse..." - Instead you can say as 'This study was conducted in greenhouse conditions where the water provided to the plants will not be lost as the polybag do not have holes in it.

 "Another factor that can be used as a cause of high chlorophyll levels is duration growth (perennial plants hundreds of years old" - What do you mean? If the life is more, you will find more chlorophyll? Chlorophyll content of each clone varies irrespective of its life duration whether it is 100 years or one year, as you have rightly mentioned in the last line of the same para that plants shed leaves naturally every year. So, please change the meaning of the above sentence suitably.

SOD

 The reason for no much change in SOD activity across the treatments is because the light factor you have used in this study is not sufficient to make damage in these plants, so is the normal SOD levels. When more light under stress, more ROS production could have been seen in 30 % water treatment I believe. So, SOD cannot be used as drought marker becomes unacceptable.

In POD para: "clones IRR 425, IRR 428, IRR 429, IRR 434, IRR 440, RRIC 100, and BPM 24." 'as scions' is not needed as when you say clone, that itself denotes it.

 "Based on the findings, several analyses have been carried out on physiological characteristics to determine the effects of water content on a greenhouse scale." - Instead you can take it to the Conclusion paragraph and explain like below: 

 'Based on the physiological and biochemical parameters made in this investigation to determine the effect of water content in a greenhouse scale, the findings indicate that only few parameters are reliable as indicators of drought stress tolerance. For e.g. the parameters such as total sugar content and proline were exhibiting strong correlation with drought stress tolerance in the IRR 400 series. They were produced at higher levels in plants treated with lesser water content. However, the parameters such as chlorophyll a, b, and total, H _2_O _2_, APX SOD, and POD did not show any correlation to used as drought tolerance indicators in this experiment.'

 "(we cannot generalize the statement that these parameters should not be used as markers for drought stress tolerance in rubber trees as mentioned in the concluding line)." - You have to be very cautions when you make such statements. You can only say with much precaution that this study did not show any correlation.

 These are the main corrections I found crucial to improvise the manuscript. In general, the parameters studied are appropriate, but I doubt the light availability in this study might not have been sufficient to induce much damage to the leaves to cause chlorophyll degradation and ROS production. You have not given any information about the PAR or the light conditions you provided to the plants though it was enquired during the first review. Probably you might have to increase the PAR next time.

 I wish all the best with the research in this line. I hope with these corrections your manuscript would become much better and meaningful. Good luck.

Is the work clearly and accurately presented and does it cite the current literature?

Partly

If applicable, is the statistical analysis and its interpretation appropriate?

No

Are all the source data underlying the results available to ensure full reproducibility?

Partly

Is the study design appropriate and is the work technically sound?

Yes

Are the conclusions drawn adequately supported by the results?

Partly

Are sufficient details of methods and analysis provided to allow replication by others?

Partly

Reviewer Expertise:

Plant Physiology, Molecular Biology, Plant Breeding, etc.

I confirm that I have read this submission and believe that I have an appropriate level of expertise to confirm that it is of an acceptable scientific standard, however I have significant reservations, as outlined above.

# Reviewer report: Reviewer response for version 2

**DOI:** 10.5256/f1000research.152374.r188304

**Published:** 2023-08-09

**Authors:** Maryam Nazari

**Affiliations:** 1 Department of Agronomy and Plant Breeding, Faculty of Agriculture, Bu-Ali Sina University, Hamadan, Iran

Thanks to the authors for revising the article accordingly. I have reviewed the new version and I think the author's response and the changes made to improve the article are appropriate.

Is the work clearly and accurately presented and does it cite the current literature?

Yes

If applicable, is the statistical analysis and its interpretation appropriate?

Partly

Are all the source data underlying the results available to ensure full reproducibility?

Yes

Is the study design appropriate and is the work technically sound?

Yes

Are the conclusions drawn adequately supported by the results?

Yes

Are sufficient details of methods and analysis provided to allow replication by others?

Partly

Reviewer Expertise:

NA

I confirm that I have read this submission and believe that I have an appropriate level of expertise to confirm that it is of an acceptable scientific standard.

# Reviewer report: Reviewer response for version 1

**DOI:** 10.5256/f1000research.142106.r164844

**Published:** 2023-04-25

**Authors:** Maryam Nazari

**Affiliations:** 1 Department of Agronomy and Plant Breeding, Faculty of Agriculture, Bu-Ali Sina University, Hamadan, Iran

The authors have done considerable work and this manuscript could be indexed and would expand our knowledge. However, I list some points that the authors should consider to improve the revised version:

**Methods**

- There is no explanation about the stages of growth and development of the clones in this manuscript.

**Results**

- According to table 1, the IRR 425 clone had the lowest total sugar content four times not three times.
- According to table 3, the RRIC 100 had the highest total sugar in two observations (third and sixth) not third and fourth.

**Discussion**

- The IRR 425 clone had the highest proline levels in three observations not four.
- Please be very careful about using very different experimental protocols to explain your results.  The key to making your paper compelling is to tie the previous research and the broader literature to your specific research (where the results agree or disagree) while avoiding speculating too much on traits that you did not measure. Please revise the following paragraph;
  "In this study, it was seen that chlorophyll a, b, and total levels at 30% were higher than 90%. This is presumably because the rubber plant is an annual plant that is able to adapt to water shortages as its root structure, taproot, grows deeper to find water further from the soil surface…"

Is the work clearly and accurately presented and does it cite the current literature?

Yes

If applicable, is the statistical analysis and its interpretation appropriate?

Partly

Are all the source data underlying the results available to ensure full reproducibility?

Yes

Is the study design appropriate and is the work technically sound?

Yes

Are the conclusions drawn adequately supported by the results?

Yes

Are sufficient details of methods and analysis provided to allow replication by others?

Partly

Reviewer Expertise:

Plant breeding

I confirm that I have read this submission and believe that I have an appropriate level of expertise to confirm that it is of an acceptable scientific standard, however I have significant reservations, as outlined above.

# Response

**Published:** 2023-06-30

**Authors:** Mohammad Basyuni

**Affiliations:** Universitas Sumatera Utara, Indonesia

The authors have done considerable work and this manuscript could be indexed and would expand our knowledge. However, I list some points that the authors should consider to improve the revised version:

**Response (R): We thank Reviewer for her critical reading on our manuscript and for providing us valuable suggestions. This has helped us greatly in improving this study, we have revised and addressed all of the suggestions and concerns. In the following pages we address the reviewers’ comments point by point**

 Methods

 There is no explanation about the stages of growth and development of the clones in this manuscript.

**R:** **Thank you for your suggestion. New sentences have been added to incorporate reviewer’s comments.**

**The plants age is 6-12 months planted in polybags measuring 50 x 60 cm filled with 30 kg ultisol soil. The samples used were normal leaves and not affected by leaf fall disease disease (oidium, colletotrichum, corynespora and pestalotiosis). The leaves were taken between at 08.00-09.00 o’clock in the morning. The average temperature in the greenhouse during the six months observation was 25.9 ^o^C (morning), 33.9 ^o^C (days) and 30.4 ^o^C (afternoon).** **The average temperature outside the greenhouse was 22.9 ^o^C (morning), 28.1 ^o^C (days) and 25.5 ^o^C (afternoon).** **The** **average humidity is 92.6% (morning), 62.9% (days) and 69.2% (afternoon).**

 Results

 According to table 1, the IRR 425 clone had the lowest total sugar content four times not three times.

**R:** **Thank you for the suggestion (correction made)**

 According to table 3, the RRIC 100 had the highest total sugar in two observations (third and sixth) not third and fourth.

**R: Correction made**

 Discussion

 The IRR 425 clone had the highest proline levels in three observations, not four.

**R:** **Thank you for the correction. Done as recommended.**

 Please be very careful about using very different experimental protocols to explain your results.  The key to making your paper compelling is to tie the previous research and the broader literature to your specific research (where the results agree or disagree) while avoiding speculating too much on traits that you did not measure. Please revise the following paragraph;

**R: Correction made**

 "In this study, it was seen that chlorophyll a, b, and total levels at 30% were higher than 90%. This is presumably because the rubber plant is an annual plant that is able to adapt to water shortages as its root structure, taproot, grows deeper to find water further from the soil surface…"

 In this study, it was seen that chlorophyll a, b, and total levels at 30% were higher than at 90%.  This is presumably because the rubber plant is a perennial plant that is able to adapt when water conditions are on a small scale due to the root structure of the rubber plant It has a taproot that will grow even deeper to find water farther from the ground. In addition, when stressed, the lateral roots will grow even more to take advantage of the existing water on the surface.

**R:** **Thank you for the suggestion (correction made)**

**Khayatnezhad, et al, 2023 have reported that genotypes corn BC678 and BC404 have the highest chlorophyll index and the amount of yield and are the most resistant genotypes to drought (reference no.38).**

# Response

**Published:** 2023-10-06

**Authors:** Mohammad Basyuni

**Affiliations:** Universitas Sumatera Utara, Indonesia

APPROVED WITH RESERVATIONS

**Response (R):** **We thank Reviewer for her critical reading on our manuscript and for providing us valuable suggestions. This has helped us greatly in improving this study, we have revised and addressed all of the suggestions and concerns i** **n the following pages .**

 Introduction second para

 1. One of the primary sources of natural rubber is - please insert with 'producing plants' was found in the Amazon..

 R: Correction made

 2. northeastern states of India - does not experience drought, instead say the 'Central India'.

 R: Correction made

 Introduction third para

 3. "Based on bio-informatics, there are 20 proteins related to drought stress in rubber..." This can be written as, 'Pasaribu et al., reported...20 drought stress related proteins...', otherwise it would mean that there are only 20 drought related proteins found in Hevea.

 R: Done as recommended

 Methods, Stages of growth and development.....

 1. second line: insert space between "...Corynespora and"

 R: Correction made

 2. Change the sentence to 'The leaves were taken between 8.00 AM and 9.00 AM'. Morning is not needed as AM is given.

 R: Correction made

 3. Data Analysis: First line - change to 'with an interval of three weeks'.

 R: Correction made

 Results

 1. Proline unit mgg-1 should be changed to mg g-1. There should be space between the mg and g. Replace wherever it is applicable as this is found throughout the manuscript.

 R: Correction made

 2. 'chlorophyll (microgram) ug mg-1'

 R: Correction made

 3. Instead of "Chlorophyll total change" it to 'Total Chlorophyll content'

 R: Correction made

 4. Ascorbate peroxidase/APX (units mg-1) - space inserted.

 R: Correction made

 5. SOD. POD - also (units mg-1)

 R: Correction made

 Discussion - First Paragraph: Please recast it as it does not convey the message properly.

 R: Correction made

 For e.g. You can try like below.

 'Plants respond and adapt to the change in environmental conditions such as drought stress by altering its physiological and biochemical activities to overcome such stressful situations.'

 R: Correction made

 The second and third line are mere repetitions of the first line which you can avoid.

 R: Correction made

 (changing the sentence according to the reviewer's suggestions)

 Second para: the first line does not convey the message properly.

 I think it should have been - 'The plants respond to the environmental stress by altering its physiological activities so as to maintain its growth and development without succumbing to the stress by producing compounds that render tolerance.'

 R: Correction made

 In third para, third line:

 "Each clone showed its ability to produce total sugar content when stressed." - what do you mean by this. It is obvious that even without stress, total sugar content will always be there. Do you want to say that 'Each clone had altered levels of total sugar content under stress conditions?

 R: Correction made

 Fourth para, third line from the bottom:

 "This shows that the clonal factor still has to be tested in other environments against drought stress." - What do you mean by this sentence? How do you want to test in other environments against drought stress?

 R: Correction made

 6th para...

 "This is presumably because the rubber plant is an perennial plant..." - 'an' is not required, instead just use 'a' only.

 R: Correction made

 (literature 39) /additional literature

 second line

 "It has a taproot that will grow even deeper to find water farther from the ground." - Generally the bud-grafted plants do not have tap root that can go deeper to find water. The root system will be to a length of about one meter only in the ground below. It only uses the lateral roots to find water on the top layer. So how will you substantiate your statement?

 7th para:

 "The condition of this research is a scale research greenhouse..." - Instead you can say as 'This study was conducted in greenhouse conditions where the water provided to the plants will not be lost as the polybag do not have holes in it.

 "Another factor that can be used as a cause of high chlorophyll levels is duration growth (perennial plants hundreds of years old" - What do you mean? If the life is more, you will find more chlorophyll? Chlorophyll content of each clone varies irrespective of its life duration whether it is 100 years or one year, as you have rightly mentioned in the last line of the same para that plants shed leaves naturally every year. So, please change the meaning of the above sentence suitably.

 R: Correction made

 SOD

 The reason for no much change in SOD activity across the treatments is because the light factor you have used in this study is not sufficient to make damage in these plants, so is the normal SOD levels. When more light under stress, more ROS production could have been seen in 30 % water treatment I believe. So, SOD cannot be used as drought marker becomes unacceptable.

 R: Correction made

 In POD para: "clones IRR 425, IRR 428, IRR 429, IRR 434, IRR 440, RRIC 100, and BPM 24." 'as scions' is not needed as when you say clone, that itself denotes it.

 "Based on the findings, several analyses have been carried out on physiological characteristics to determine the effects of water content on a greenhouse scale." - Instead you can take it to the Conclusion paragraph and explain like below:

 R: Correction made

 Changing the sentence as suggestion reviewer

 'Based on the physiological and biochemical parameters made in this investigation to determine the effect of water content in a greenhouse scale, the findings indicate that only few parameters are reliable as indicators of drought stress tolerance. For e.g. the parameters such as total sugar content and proline were exhibiting strong correlation with drought stress tolerance in the IRR 400 series. They were produced at higher levels in plants treated with lesser water content. However, the parameters such as chlorophyll a, b, and total, H2O2, APX SOD, and POD did not show any correlation to used as drought tolerance indicators in this experiment.'

 Thank you for your suggestions.

 "(we cannot generalize the statement that these parameters should not be used as markers for drought stress tolerance in rubber trees as mentioned in the concluding line)." - You have to be very cautions when you make such statements. You can only say with much precaution that this study did not show any correlation.

 These are the main corrections I found crucial to improvise the manuscript. In general, the parameters studied are appropriate, but I doubt the light availability in this study might not have been sufficient to induce much damage to the leaves to cause chlorophyll degradation and ROS production. You have not given any information about the PAR or the light conditions you provided to the plants though it was enquired during the first review. Probably you might have to increase the PAR next time.

 I wish all the best with the research in this line. I hope with these corrections your manuscript would become much better and meaningful. Good luck.

# Reviewer report: Reviewer response for version 1

**DOI:** 10.5256/f1000research.142106.r161733

**Published:** 2023-04-06

**Authors:** Mohamed sathik Thirruvithamkottil

**Affiliations:** 1 Rubber Research Institute of India, Rubber Board, Kottayam, Kerala, India

Title: it should be Physiological characteristics of IRR 400 series rubber clones (Hevea brasiliensis Muell. Arg.) under drought stress

**Abstract:**

**Background**

 Second line: Plants survival – should be changed to - plant’s ; Triggering of specific signaling pathways and no comma mark (,) is needed  before ‘and’

 7 ^th^ line: A tolerant rubber clone in a dry area is the right approach. Should have been ‘Growing or planting tolerant rubber clones in drought prone areas would be more appropriate.

 The present study **is** aimed (is missing)…… to obtain character selection is not the appropriate way of communication… should have been ‘to identify drought tolerant traits in order to select or identify drought-tolerant clones at juvenile stage itself.

**Introduction**

 The second line of third paragraph says ‘results in reduced growth rate, **stem elongation**, leaf senescence and **stomatal opening.’  **In contradiction, in the fourth line it says ‘the fastest response to a (usage of ‘a’  is unnecessary here) water deficit is the stomatal closure to protect plants from water shortages’.  The authors have to be careful and explain things in logic and avoid making contradictory statements like this.

 Usage of ‘plays’ and ‘enables’ with plural factors mentioned in the last two lines of third para is irrelevant. Instead it should be play and enable. The English language usage needs to be verified.

 ‘Stem elongation’ in the second line of third paragraph and ‘inhibited growth’ in the first line of fourth paragraph are contradictory and needs to be corrected.

**Methods**

 Details regarding points such as 1) Age of the plant, 2) whether field grown or polybag grown is not mentioned, sampling (whether it is leaf or what) at what stage, size of the plant (height/girth), what was the light level and the temperature, on which day the samples were collected for the assay, etc are missing and have to be provided to give an overall view of the experimental details to the reader.

**Results**

**Table 1, 2 and 3**

 1.‘Six observations of rubber clones’ does not convey whether it is six replications or six time period or what?

 The data of all the clones should have been given at all the time point so as to give a glimpse of the data to the reader instead of giving only the highest and lowest that too splitting into three tables. Instead it should have been furnished in one table so that the reader can appreciate the whole data and can comprehend the take home message out of it.

 Similarly, Table 4,5 and 6 (of Proline) should be combined and all the figures should be given.

 Table 7,8 and 9 (of chlorophyll a) – should be combined as well and all the figures should be given.

 Table 10,11and 12 (of chlorophyll b)  – all should be combined and all the figures should be given.

 Table 13, 14 and 15 – should be combined and all the figures should be given.

 Table 16, 17 and 18 - should be combined and all the figures should be given.

 Similarly for all the tables of APX, SOD, POD.

**Discussion**

 The very first sentence needs logical correction. The sentences following that are not coherent and are like bit by bit messages put together without any continuity which should be addressed appropriately.

 Second paragraph, fourth line says’ RRIC 100 is a dry tolerant clone in the field’.  Does the author mean that it is drought tolerant because both are different.  Drying is desiccation where as drought is a complete water deficit stress which is different from drying alone. Hence, utmost care should have been taken while drafting the manuscript.

 Last sentence of second paragraph says ‘similar results of increased total sugar accumulation have been produced’, instead it would have been better if writing ‘observed or reported’.

 In third paragraph the statement that ‘Increased proline content in drought stressed plants – provides high energy to increase plant growth in water-deficit conditions’ needs to be relooked into as does it really provide high energy or induces plant growth through other mechanisms.

 Chlorophyll paragraph from the end of page 17 over to page 18, it says that ‘the rubber plant in an annual plant that is able to adapt to water shortages as its root structure, taproot, grows deeper to find water from the soil surface’. I wonder how the authors presumed that rubber is an annual plant while it being perennial. Moreover, it is a bud-grafted clonal material (of each varuet/genotype) which does not have a tap root to mine water from the deeper soil as mentioned in the above sentence. I am sure the authors have to re-check such misleading statements that are against the fact and make logical corrections in it.

 From the same paragraph, for the first time we can see mentioning of polybags where it is also mentioned that growing media (that means is it not soil?) and no hole in the polybags (which are generally made with holes to drain excess water during its pre-treatment conditions.

 The 6 ^th^ paragraph of p18, starting with ‘Thought the SOD levels……. (it should have been ‘Though’) has contradictory statements like ‘showed significant effect due to water content in the first line while in the fourth line, it says ‘low levels in the observations and therefore cannot be used as a marker of tolerance.  This kind of contradictory statements have to be looked into and corrected appropriately.

 At the end of the discussion portion, it is mentioned that ‘Based on the findings, several analysis have been carried out on physiological characteristics to determine the **effects of water content** on a greenhouse scale’ which is mentioned in many places while I wonder should it be mentioned like ‘effect of drought stress’?

**Conclusions**

 The first sentence says ‘The tolerance ability of the IRR 400 series rubber clones to drought stress ( **while it was mentioned as ‘effect of water content’ in many other places**) was determined by observing the two characteristics  of total sugar and proline levels (while they have recorded the other parameters also) and I wonder why they restrict to only two parameters here. They continue that ‘Furthermore, chlorophyll a, b and total H2O2, APX SOD and POD should not be used as markers for drought stress tolerance in rubber trees. The authors should bear in mind that they have done this study in polybag plants in green house condition and with this limited study, they cannot declare that these cannot be used as markers for drought stress tolerance in trees.

 So, I strongly feel that the authors should revisit this paper and edit it to make it perfect both technically as well as English language wise. Results and Discussion needs thorough modifications and the tables should be combined with all the data points statistically analyzed rather than providing only the extreme values.

Is the work clearly and accurately presented and does it cite the current literature?

Partly

If applicable, is the statistical analysis and its interpretation appropriate?

No

Are all the source data underlying the results available to ensure full reproducibility?

Partly

Is the study design appropriate and is the work technically sound?

Yes

Are the conclusions drawn adequately supported by the results?

Partly

Are sufficient details of methods and analysis provided to allow replication by others?

Partly

Reviewer Expertise:

Plant Physiology, Molecular Biology, Plant Breeding, etc.

I confirm that I have read this submission and believe that I have an appropriate level of expertise to confirm that it is of an acceptable scientific standard, however I have significant reservations, as outlined above.

# Response

**Published:** 2023-06-30

**Authors:** Mohammad Basyuni

**Affiliations:** Universitas Sumatera Utara, Indonesia

**Response (R): We thank Reviewer 1 for his comments on the merit of our work and for providing constructive comments and suggestions to improve our manuscript. We have revised the manuscript by addressing suggestions as follows in bold font:**

 Title: it should be Physiological characteristics of IRR 400 series rubber clones ( *Hevea brasiliensis *Muell. Arg.) under drought stress

**R: Correction made,  please refer to page 1**

 Abstract:

 Background

 Second line: Plants survival – should be changed to - plant’s ; Triggering of specific signaling pathways and no comma mark (,) is needed  before ‘and’

**R: Thank you for your correction. Done as recommendation, please refer to page 2**

 7th line: A tolerant rubber clone in a dry area is the right approach. Should have been ‘Growing or planting tolerant rubber clones in drought prone areas would be more appropriate.

**R: Correction made, see page 2**

 The present study is aimed (is missing)…… to obtain character selection is not the appropriate way of communication… should have been ‘to identify drought tolerant traits in order to select or identify drought-tolerant clones at juvenile stage itself.

**R: Thank you for the correction. The present study aimed to determine the mechanism of drought-tolerant clones, based on physiological characteristics, to identify drought tolerant traits in order to select drought tolerant clones at juvenile stage. Please refer to page 2**

 Introduction

 The second line of third paragraph says ‘results in reduced growth rate, stem elongation, leaf senescence and stomatal opening.’  In contradiction, in the fourth line it says ‘the fastest response to a (usage of ‘a’  is unnecessary here) water deficit is the stomatal closure to protect plants from water shortages’.  The authors have to be careful and explain things in logic and avoid making contradictory statements like this.

**R: Thank you for your correction. The word stem elongation and stomatal has been removed to make clarity and readability. Please refer to page 2**

 Usage of ‘plays’ and ‘enables’ with plural factors mentioned in the last two lines of third para is irrelevant. Instead it should be play and enable. The English language usage needs to be verified.

**R: Thank you for your suggestions. Correction made. Please refer to page 3**

 ‘Stem elongation’ in the second line of third paragraph and ‘inhibited growth’ in the first line of fourth paragraph are contradictory and needs to be corrected.

**R: Thank you for your suggestions. The initial response to drought stress is loss of turgor pressure, which results in reduced growth rate, rapid stem growth, and leaf senescence.  “Stem elongation” and “stomatal opening” have been deleted.**

 Methods

 Details regarding points such as 1) Age of the plant, 2) whether field grown or polybag grown is not mentioned, sampling (whether it is leaf or what) at what stage, size of the plant (height/girth), what was the light level and the temperature, on which day the samples were collected for the assay, etc are missing and have to be provided to give an overall view of the experimental details to the reader.

**R: Thank you for suggestions. We have added new sentences to incorporate your suggestions: Stages of growth and development of the clones**

**The plants age is 6-12 months planted in polybags measuring 50 x 60 cm filled with 30 kg ultisol soil. The samples used were normal leaves and not affected by leaf fall disease disease (Oidium, Ccolletotrichum, Ccorynespora and Pestalotiopsis). The leaves were taken between at 08.00-09.00 o’clock in the morning. The average temperature in the greenhouse during the six months observation was 25.9 ^o^C (morning), 33.9 ^o^C (days) and 30.4 ^o^C (afternoon). The average temperature outside the greenhouse was 22.9 ^o^C (morning), 28.1 ^o^C (days) and 25.5 ^o^C (afternoon). The average humidity is 92.6% (morning), 62.9% (days) and 69.2% (afternoon). Please refer to page 4.**

 Results

 Table 1, 2 and 3

 1.‘Six observations of rubber clones’ does not convey whether it is six replications or six time period or what?

**R: Thank you for the correction (correction made as six time period): Observations of physiological characters were observed 6 times with a time distance 21 days between observations. The total samples observed were 63 samples consisting 7 types of clones x 3 types of water concentration and 3 replications. Please refer to Tables 1-9.**

 The data of all the clones should have been given at all the time point so as to give a glimpse of the data to the reader instead of giving only the highest and lowest that too splitting into three tables. Instead it should have been furnished in one table so that the reader can appreciate the whole data and can comprehend the take home message out of it.

**R: Thank you for your suggestions**. **Done as recommended.**

 Similarly, Table 4, 5 and 6 (of Proline) should be combined and all the figures should be given.

**R: Thank you for the suggestion. Correction made.**

 Table 7,8 and 9 (of chlorophyll a) – should be combined as well and all the figures should be given.

**R: Thank you for the advice. Done as recommended.**

 Table 10,11and 12 (of chlorophyll b)  – all should be combined and all the figures should be given.

**R: Thank you for the suggestion. Correction made.**

 Table 13, 14 and 15 – should be combined and all the figures should be given.

**R: Thank you for the advice. Done as recommended.**

 Table 16, 17 and 18 - should be combined and all the figures should be given.

**R: Thank you for the advice. Correction made.**

 Similarly for all the tables of APX, SOD, POD.

**R: Thank you for the advice. Correction made.**

 Discussion

 The very first sentence needs logical correction. The sentences following that are not coherent and are like bit by bit messages put together without any continuity which should be addressed appropriately.

**R:** **Thank you for the correction, we have revised accordingly:  Plants respond and adapt due to environmental changes that experience continuous drought stress with appropriate physiological and biochemical changes to overcome these stressful conditions.** **Stress in plants is a physiological situation that is induced when there is a severe or constant change in the environment or when aggressive normal conditions, changing the physiological and adaptive patterns of plants.** **All these environmental modifications produce physiological reactions in cells of genetic origin.**

 Second paragraph, fourth line says’ RRIC 100 is a dry tolerant clone in the field’.  Does the author mean that it is drought tolerant because both are different.  Drying is desiccation where as drought is a complete water deficit stress which is different from drying alone. Hence, utmost care should have been taken while drafting the manuscript.

**R: Thank you for the suggestion.** **There are differences in growth between areas  that have distinct rainy and dry seasons, such as in Java.** **The RRIC 100 clone has a faster growth and higher production potential compared to intolerant clones in the area such as the BPM 24 clone.**

 Last sentence of second paragraph says ‘similar results of increased total sugar accumulation have been produced’, instead it would have been better if writing ‘observed or reported’.

**R: Thank you for the suggestion** **, correction made.** **Similar results of increased total sugar accumulation have been o** **bserved.**

 In third paragraph the statement that ‘Increased proline content in drought stressed plants – provides high energy to increase plant growth in water-deficit conditions’ needs to be relooked into as does it really provide high energy or induces plant growth through other mechanisms.

**R: Thank you for the advice** **(correction made):** **similar results of increased total sugar accumulation have been o** **bserved.**

 Chlorophyll paragraph from the end of page 17 over to page 18, it says that ‘the rubber plant in an annual plant that is able to adapt to water shortages as its root structure, taproot, grows deeper to find water from the soil surface’. I wonder how the authors presumed that rubber is an annual plant while it being perennial. Moreover, it is a bud-grafted clonal material (of each varuet/genotype) which does not have a tap root to mine water from the deeper soil as mentioned in the above sentence. I am sure the authors have to re-check such misleading statements that are against the fact and make logical corrections in it.

**R: Thank you for your correction.**

**We made a mistake in mentioning that the rubber plant is annual plant, it should be a perennial plant. In this study it can be seen that the content of chlorophyll a, b, and total at 30% water content is higher than that at 90% water content.** **This is presumably because the rubber plant is an perennial plant that is able to adapt when water conditions are on a small scale due to the root structure of the rubber plant It has a taproot that will grow even deeper to find water farther from the ground.** **In addition, when stressed, the lateral roots will grow even more to take advantage of the existing water on the surface.**

**The condition of this research is a scale research greenhouse, the water given to the planting medium will not be lost down because the planting medium is designed not to have holes or gaps where the water comes out.** **Besides that, the polybag surface is also covered by plastic to minimize the occurrence of evapotranspiration from the planting medium.** **Matter Another factor that can be used as a cause of high chlorophyll levels is duration growth (perennial plants hundreds of years old), plant size (large), ability to adjust osmolyte content (sugar, proline, glycine betaine, ABA, ethylene, and others), and the nature of the rubber plant itself (plants that will shed leaves naturally every year).**

 From the same paragraph, for the first time we can see mentioning of polybags where it is also mentioned that growing media (that means is it not soil?) and no hole in the polybags (which are generally made with holes to drain excess water during its pre-treatment conditions.

**R: Thank you for the correctiom.**

**It means that the water is added to soil which is placed in polybag covered with more transparent paste which has no gaps.** **So that the water will remain in the polybag.**

 The 6th paragraph of p18, starting with ‘Thought the SOD levels……. (it should have been ‘Though’) has contradictory statements like ‘showed significant effect due to water content in the first line while in the fourth line, it says ‘low levels in the observations and therefore cannot be used as a marker of tolerance.  This kind of contradictory statements have to be looked into and corrected appropriately.

**R: Thank you for the suggestion.**

**The SOD result should be different between the water content.** **But in our research, the same results were obtained between different water content.**

 At the end of the discussion portion, it is mentioned that ‘Based on the findings, several analysis have been carried out on physiological characteristics to determine the effects of water content on a greenhouse scale’ which is mentioned in many places while I wonder should it be mentioned like ‘effect of drought stress’?

**R: Thank you for the suggestion. Correction made.**

 Conclusions

 The first sentence says ‘The tolerance ability of the IRR 400 series rubber clones to drought stress (while it was mentioned as ‘effect of water content’ in many other places) was determined by observing the two characteristics of total sugar and proline levels (while they have recorded the other parameters also) and I wonder why they restrict to only two parameters here. They continue that ‘Furthermore, chlorophyll a, b and total H2O2, APX SOD and POD should not be used as markers for drought stress tolerance in rubber trees. The authors should bear in mind that they have done this study in polybag plants in green house condition and with this limited study, they cannot declare that these cannot be used as markers for drought stress tolerance in trees.

**R: Thank you for the suggestion.**

**The water content with a concentration of 30% is considered to reflect dry conditions. So it is referred as drought stress. For the observational characters, two characters were obtained, namely total sugar and proline which showed a significant effect from the 6 time observations.** **As for the other observational characters (Chlorophyll a, b, total, APX, H2O2, SOD and POD) they still showed the same effect when treated with several water concentrations.**

 So, I strongly feel that the authors should revisit this paper and edit it to make it perfect both technically as well as English language wise. Results and Discussion needs thorough modifications and the tables should be combined with all the data points statistically analyzed rather than providing only the extreme values.

**R:** **Thank you for the suggestions. Revision made.**
